# Gαi1/3 mediate Netrin-1-CD146-activated signaling and angiogenesis

**DOI:** 10.7150/thno.80749

**Published:** 2023-04-17

**Authors:** Ya Li, Jin-long Chai, Xin Shi, Yu Feng, Jia-jun Li, Li-na Zhou, Cong Cao, Ke-ran Li

**Affiliations:** 1 Clinical Research Center of Neurological Disease, The Second Affiliated Hospital of Soochow University and North District, The Municipal Hospital of Suzhou, Gusu School, Nanjing Medical University, Suzhou, China.; 2 Department of Endocrinology, The Second Affiliated Hospital of Soochow University, Suzhou, China.; 3 The Affiliated Eye Hospital, Nanjing Medical University, Nanjing, China.; 4 Department of Radiotherapy and Oncology, Kunshan First People's Hospital Affiliated to Jiangsu University, Kunshan, China.

**Keywords:** Netrin-1, Angiogenesis, Gαi1 and Gαi3, CD146, Diabetic Retinopathy

## Abstract

Netrin-1 binds to the high-affinity receptor CD146 to activate downstream signaling and angiogenesis. Here, we examine the role and underlying mechanisms of G protein subunit alpha i1 (Gαi1) and Gαi3 in Netrin-1-induced signaling and pro-angiogenic activity. In mouse embryonic fibroblasts (MEFs) and endothelial cells, Netrin-1-induced Akt-mTOR (mammalian target of rapamycin) and Erk activation was largely inhibited by silencing or knockout of Gαi1/3, whereas signaling was augmented following Gαi1/3 overexpression. Netrin-1 induced Gαi1/3 association with CD146, required for CD146 internalization, Gab1 (Grb2 associated binding protein 1) recruitment and downstream Akt-mTOR and Erk activation. Netrin-1-induced signaling was inhibited by CD146 silencing, Gab1 knockout, or Gαi1/3 dominant negative mutants. Netrin-1-induced human umbilical vein endothelial cell (HUVEC) proliferation, migration and tube formation were inhibited by Gαi1/3 short hairpin RNA (shRNA), but were potentiated by ectopic Gαi1/3 overexpression. *In vivo*, intravitreous injection of Netrin-1 shRNA adeno-associated virus (AAV) significantly inhibited Akt-mTOR and Erk activation in murine retinal tissues and reduced retinal angiogenesis. Endothelial knockdown of Gαi1/3 significantly inhibited Netrin1-induced signaling and retinal angiogenesis in mice. *Netrin-1* mRNA and protein expression were significantly elevated in retinal tissues of diabetic retinopathy (DR) mice. Importantly, silence of Netrin-1, by intravitreous Netrin-1 shRNA AAV injection, inhibited Akt-Erk activation, pathological retinal angiogenesis and retinal ganglion cells degeneration in DR mice. Lastly, Netrin-1 and CD146 expression is significantly increased in the proliferative retinal tissues of human proliferative diabetic retinopathy patients. Together, Netrin-1 induces CD146-Gαi1/3-Gab1 complex formation to mediate downstream Akt-mTOR and Erk activation, important for angiogenesis *in vitro* and *in vivo*.

## Introduction

Vascular dysfunction participates in the pathogenesis and progression of many human diseases, including heart failure, diabetes, cancer, retinal and many others 1-5. In the absence of stimulation, endothelial cells, located in the lumen of blood vessels 4, 5, rarely proliferate and remain in a quiescent state 4, 5. Nutrient deprivation or growth factor stimulation [*e.g.* vascular endothelial growth factor (VEGF)] can rapidly initiate angiogenesis to form new vessels 4-8.

Netrin-1 promotes neuronal axon outgrowth during the development of central nervous system 9. Netrin-1 has an N-terminal laminin repeat, three cysteine-rich epidermal growth factormodules and a Netrin-like domain 9. Netrin-1 can either promote or suppress endothelial cell activation and angiogenesis 10-13, depending on its concentration and the receptor it binds 10-14. Yu *et al.,* reported that at low concentrations (<μg/mL) of Netrin-1 promoted migration, invasion and tube formation of human umbilical vein endothelial cells (HUVECs), and increased retinal neovascularization when injected intravitreally 15. At higher concentrations, however, Netrin-1 was shown to suppress angiogenesis *in vitro* and *in vivo* 15.

Netrin-1 binds the cognate receptor Unc-5 Netrin receptor B (UNC5B) in endothelial cells, causing the anti-angiogenic effect 10. Tu *et al.,* have discovered CD146 (melanoma cell adhesion molecule, MCAM) as a functional receptor of Netrin-1 in endothelial cells 14. CD146 binds low concentrations of Netrin-1 at high affinity, activating endothelial cells to promote angiogenesis 14. Conditional knockdown of CD146 or a specific anti-CD146 antibody prevented Netrin-1-induced angiogenesis 14.

G protein subunit alpha i (Gαi) proteins have three members: Gαi1, Gαi2 and Gαi3. Gαi protein binding to G protein coupled receptors inhibits adenylyl cyclase activation and depletes cyclic AMP (cAMP) 16, 17. Our group and others have defined an essential role of Gαi1 and Gαi3 in mediating signaling transduction by multiple receptor tyrosine kinases (RTKs) 18-27. Following epidermal growth factor (EGF) stimulation, Gαi1/3 and the adaptor protein Grb2 associated binding protein 1 (Gab1) associate with the EGFR to promote downstream Akt-mammalian target of rapamycin(mTOR) 26, 27. Similarly, keratinocyte growth factor (KGF) stimulation led to the formation of a KGFR-Gαi1/3-Gab1 complex, mediating downstream Akt-mTOR activation 25. Gαi1/3 also promoted endocytosis of VEGF-activated VEGFR and brain-derived neurotrophic factor (BDNF)-activated TrkB, thereby transducing downstream signaling 22, 23. YME1 Like 1 ATPase was found to increase Gαi1 expression and downstream Akt activation, promoting glioma cell growth 19. Our recent study showed that phosphoenolpyruvate carboxykinase 1 (PCK1) associated with GATA binding protein 4 to promote *Gαi3* transcriptional and expression, thus increasing Akt-mTOR activation in endothelial cells 18.

Our group and others have also characterized the role of Gαi1/3 proteins in mediating signaling by non-RTK receptors. Gαi1/3 immunoprecipitated with interleukin-4 (IL-4)-stimulated IL-4Rα, promoting IL-4Rα endosome translocation and downstream Gab1-Akt activation in macrophages 28. R-spondin3 induced Gαi1/3 association with leucine-rich repeat G protein-coupled receptor 4 and Gab1 to mediate downstream Akt-mTOR activation 29. Here we explore the possible involvement of Gαi1/3 in Netrin-1-activated signaling and angiogenesis. We found that Netrin-1 induces CD146 and Gαi1/3 association to mediate downstream Akt-mTOR and Erk activation, thereby promoting angiogenesis *in vitro* and *in vivo*.

## Methods

**Reagents.** Polybrene, LY294002, PD98059 and puromycin were provided by Sigma-Aldrich (St. Louis, MO). The antibodies utilized in the present study were listed in **Table 1**. Reagents for cell culture, including serum, medium, and antibiotics, were from Gibco-BRL (Suzhou, China).

**Cells.** The wild-type (WT) mouse embryonic fibroblasts (MEFs), Gαi1 plus Gαi3 double knockout (“Gαi1/3 DKO”) MEFs, Gαi1, Gαi2 or Gαi3 single knockout (SKO) MEFs, WT and Gab1 KO MEFs were described in detail in our previous studies 21-23, 25, 26, 29. Culturing of primary HUVECs was reported previously 18, 22, 30.

**Genetic modifications of Gαi1/3.** Genetic modifications of Gαi1/3 by viral constructs, including Gαi1/3 shRNA (short hairpin RNA), overexpression, knockout (KO), or dominant negative mutations, in MEFs and HUVECs, were reported in detail in our previous studies 22, 23, 29. Stable cells were always selected by puromycin-containing complete medium and Gαi1/3 expression in the stable cells was verified by quantitative real-time PCR (qRT-PCR) and Western blotting assays 20, 22, 23, 29.

**CD146 and Netrin-1 shRNA**. The generation of lentivirus-packed CD146 shRNA sequence (Genechem) in the GV248 vector (hU6-MCS-CBh-IRES-puromycin) was reported previously 18. Virus infection and stable cells formation were described previously 18. For *in vivo* studies, the Netrin-1 shRNA sequence (Genechem) was inserted into an adeno-associated virus 5 (AAV5) construct. These constructs were transfected to HEK-293 cells, generating adenovirus (AAV). The virus was then intravitreally injected to the mice.

**Tube formation.** The basement membrane matrix (BD Biosciences, Shanghai, China) was added to the 24-well plates for 30 min. HUVECs (at 1.0 × 10^5^ cells per well) were inoculated onto the pre-coated plates and incubated in the humidified incubator setting at 37°C and 5% CO_2_ for 16h. A Zeiss microscope was used to take photographs of the tube formation. The average number of formed tubes was recorded.

**5-ethynyl-2'-deoxyuridine (EdU) staining.** An EdU proliferation kit (Beyotime, Nanjing, China) was utilized to detect cell proliferation. Brief, HUVECs with different treatments were incubated with EdU solution (50 μM) for 2h at 37°C. Following fixation and washing, cells were co-stained with DAPI for another 10 min. Thereafter, cells were photographed under a fluorescent microscope (Zeiss).

***In vitro* cell migration/invasion assays.** HUVECs (at 5 × 10^4^ cells per chamber) with different treatments were added to the upper chamber of Transwell inserts with 8 μm pore (Corning, Corning, NY). The chambers were pre-coated with (testing cell invasion) or without (testing cell migration) Matrigel (BD Biosciences, Shanghai, China). The completed medium with fetal bovine serum (FBS) was added to the lower chamber. Transwell chambers were then placed in the humidified incubator setting at 37°C and 5% CO_2_. After 24 h, migrated/invaded cells were immersed into 3% paraformaldehyde for 15 min, stained with crystal violet, and photographed under a light microscope.

**qRT-PCR.** Total cellular and tissue RNA was extracted by Trizol Reagents (Thermo Fisher Scientific, Shanghai, China). Prime Script RT Reagent Kit (Takara, Kyoto, Japan) was utilized to transcribe cDNA. qRT-PCR was conducted through the ABI Prism 7900 detection system (Applied Biosystems, Shanghai, China). The reaction mixture included 2 μl cDNA template, 2 μl primers, and 10 μl of 2× SYBR Green PCR Mix (Takara). The melting temperature was always calculated. The comparative Ct method (2^-ΔΔCt^) was utilized to quantify expression of targeted mRNAs 31, with *glyceraldehyde-3-phosphate dehydrogenase* (*GAPDH*) tested as the reference gene. All the mRNA primers were from Genechem (Shanghai, China).

**Western blotting and co-immunoprecipitation (Co-IP) assays.** The cellular/tissue lysates (20-30 μg per treatment) were first separated by 10-12.5% SDS-PAGE and were then transferred to a PVDF blot. The latter was incubated in 7.5% non-fat milk for 50 min at room temperature for blocking, and the primary antibody added overnight at 4°C. After washing, the PVDF blot was incubated with secondary antibody for 30 min at room temperature. Enhanced chemiluminescence procedure was then performed to visualize the targeted protein band. For Co-IP studies, the primary antibody was first added to the total cellular lysates overnight at 4°C. The Protein A/Sepharose beads (Amersham Biosciences, Shanghai, China) were then added to extract the protein-protein complex, which was tested by Western blotting analyses.

**Phosphoproteomics.** Phosphopeptide Enrichment: SDT buffer (4% SDS, 100 mM Tris-HCl, pH 7.6) was added to the sample. The lysates were sonicated and then boiled for 15 min. After centrifuged at 14, 000g for 15 min, the supernatant was quantified with the BCA Protein Assay Kit (P0012, Beyotime). For digestion, 600 ug of proteins for each sample were reduced with 100 mM DTT for 5 min at 100 °C. Then the detergent, DTT and other low-molecular-weight components were removed using UA buffer (8 M Urea, 150 mM Tris-HCl pH 8.5). Afterwards, 100 μL iodoacetamide was added to block reduced cysteine residues and the samples were incubated for 30 min in darkness. Finally, the protein suspensions were digested with 4 μg trypsin (Promega) in 40 μL 50 mM NH_4_HCO_3_ buffer overnight at 37 °C. After trypsin digestion, peptides were desalted by C18 column and vacuum-dried. The peptides' mixture was subjected to HiSelect Fe-NTA phosphopeptide enrichment kit (Thermo Fisher Scientific, A32993) according to the protocol. The Fe-NTA eluent were dried down via vacuum centrifugation at 45 °C and then dissolved in 0.1% Formic acid buffer. LC-MS/MS analysis: Each eluent was injected for nanoLC-MS/MS analysis. The peptide mixture was loaded onto the C18-reversed phase analytical column (Thermo Fisher Scientific, Acclaim PepMap RSLC 50um X 15cm, nano viper, P/N164943) in buffer A (0.1% formic acid) and separated with a linear gradient of buffer B (80% acetonitrile and 0.1% formic acid) at a flow rate of 300 nL/min. The samples were analyzed by mass spectrometry with Q Exactive HF-X (Thermo Fisher Scientific). The scanning range of mother ion was 350-1500 m/z, and the resolution of primary mass spectrometry was 60,000. The AGC target was 3e6, with the primary maximum injection time 50 ms. The resolution of DIA (data-independent acquisition) was 30000, the AGC target was 1e6, with the normalized collision energy (NCE) was 28 eV. Data analysis: Raw Data of DIA were processed and analyzed by Spectronaut (Biognosys AG, Switzerland) with default settings. Retention time prediction type was set to dynamic iRT. Spectronaut will determine the ideal extraction window dynamically depending on iRT calibration and gradient stability. Q-value cutoff on precursor and protein level was applied 1%. After Student t test, differentially expressed proteins were filtered if their ***P*** value < 0.05 and fold change > 1.5.

**Human tissues.** As described 18, 22, six (6) different proliferative diabetic retinopathy (PDR) patients with the lensectomy plus vitrectomy surgery, as well as three (3) age-matched traumatic retinectomy patients, were enrolled. The written-informed consent was obtained from each participant. PDR patients' anterior retinal hyperplastic membrane was stripped and fresh tissue specimens were stored in liquid nitrogen. The traumatic normal retina tissues were obtained and stored in liquid nitrogen as well. The protocols were according to the principles of Declaration of Helsinki and were approved by the Ethics Board of Soochow University.

**Streptozotocin (STZ**)**-induced diabetic retinopathy (DR) mice.** C57BL/6 mice (24-28 g, aged 6-8 weeks, male) were fasted overnight and then injected intraperitoneally with streptozotocin (STZ, 60 mg/kg, Sigma) for five consecutive days. The blood glucose levels obtained from the tail vein were measured one week after the final STZ injection and then once every week. Only mice with blood glucose level > 300 mg/dL were considered diabetic and further utilized in the study. Age-matched male mice were injected intraperitoneally with equal citrate buffer as mock controls (“Mock”).

**Intravitreal injection of AAV and retinal vasculature detection.** The adult C57BL/6 mice, as reported previously 18, 29, were anesthetized, and intravitreal injection of virus was reported previously 18, 29. Approximately 0.1 μL AAV was injected into the vitreous cavity with the needle directly above the optic nerve head. The isolectin B4 (IB4) staining of retinal vasculature, retinal trypsin digestion assaying of acellular capillary formation and retinal NeuN immunofluorescence staining were reported previously 18, 29. The Institutional Animal Care and Use Committee and the Ethic Committee of Soochow University approved the protocols, and animal studies were in according to Association for Research in Vision and Ophthalmology statement.

**Statistical analysis.** Data were always with normal distribution and were expressed as mean ± standard deviation (SD). Statistical differences were calculated by Student's t test (comparing two groups) and were measured by one-way analysis of variance plus Tukey's multiple comparison test (for three or more groups comparison). Values of ***P*** < 0.05 were considered as statistically significant.

## Results

### Gαi1 and Gαi3 double knockout abolishes Netrin-1-induced Akt-mTOR and Erk activation in MEFs

**Figure 1:**
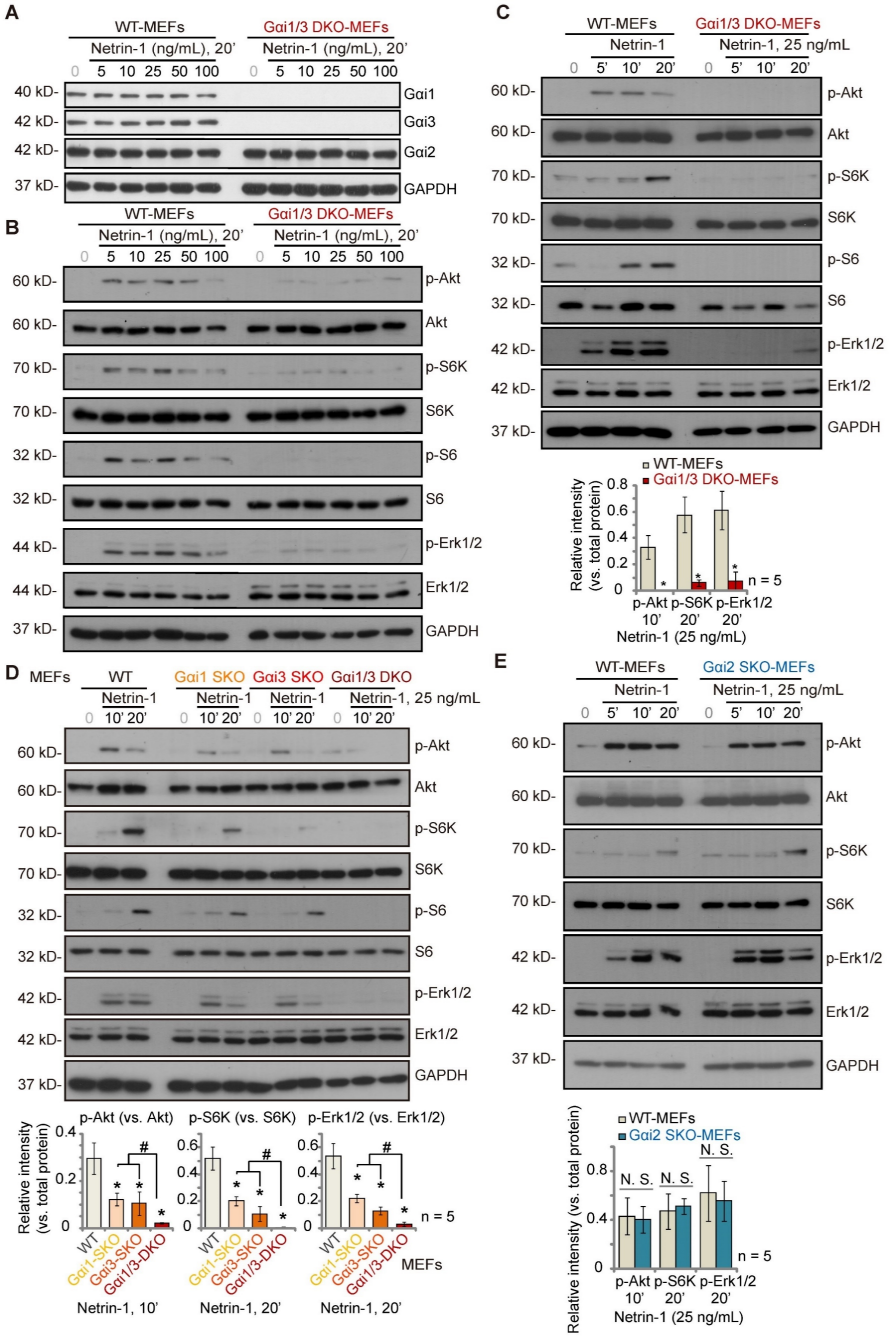
** Gαi1 plus Gαi3 double knockout abolishes Netrin-1-induced Akt-mTOR and Erk activation in MEFs**. Wild-type (WT) mouse embryonic fibroblasts (MEFs), Gαi1 plus Gαi3 double knockout (“Gαi1/3 DKO”) MEFs (**A**-**C**), Gαi1, Gαi2 or Gαi3 single knockout (SKO) MEFs (**D**-**E**) were treated with Netrin-1 at the applied concentrations for designated time periods, expression of listed proteins was shown, and protein phosphorylation was quantified (**A**-**E**). Data were expressed as mean ± standard deviation (SD, biological replicates). Quantifications were from five biological replicates (n = 5). *P < 0.05 versus “WT MEFs”. **^#^*P*** < 0.05. “N. S.” stands for non-statistical differences (***P*** > 0.05).

To explore the potential involvement of Gαi1/3 proteins in Netrin-1-activated signaling, wild-type (WT) mouse embryonic fibroblasts (MEFs) and Gαi1 plus Gαi3 double knockout (“Gαi1/3 DKO”) MEFs (see our previous studies 21-23, 25, 26, 28, 29) were treated with Netrin-1 at different concentrations (5-100 ng/mL) 14, 15. As shown, Gαi1 and Gαi3 were depleted in Gαi1/3 DKO MEFs, whereas Gαi2 protein expression was intact (Figure **1A**). Gαi1, Gαi2 and Gαi3 protein expression was unchanged following Netrin-1 treatment in WT MEFs (Figure **1A**). Treatment with Netrin-1 (5-100 ng/mL) robustly increased phosphorylation of Akt, ribosomal protein S6 kinase (S6K) and Erk1/2 in WT MEFs (Figure **1B**), indicating Akt-mTOR and Erk activation (Figure **1B**). Importantly, Netrin-1 signaling was almost completely blocked in Gαi1/3 DKO MEFs (Figure **1B**). Total Akt, S6K and Erk1/2 expression was comparable between WT and DKO MEFs (Figure **1B**).

Further studies showed that Netrin-1 (at 25 ng/mL, the dose found to induce the most significant Akt-mTOR and Erk activation, Figure **1B**) increased phosphorylation of Akt, S6K, S6 and Erk1/2 in a time-dependent manner in WT MEFs (Figure **1C**), which was abolished in Gαi1/3 DKO MEFs (Figure **1C**). Exploring the respective roles of Gαi1 and Gαi3 in Netrin-1-induced signal transduction, Netrin-1 (25 ng/mL)-induced phosphorylation of Akt, S6K, S6 and Erk1/2 was partially decreased in Gαi1 SKO or Gαi3 SKO MEFs (Figure **1D**), but completely blocked in Gαi1/3 DKO MEFs (Figure **1D**). To explore whether Gαi2 is also important for Netrin-1-induced Akt-mTOR and Erk activation, WT and Gαi2 SKO MEFs were treated with Netrin-1 (at 25 ng/mL). Figure **1E** showed that Netrin-1-induced phosphorylation of Akt, S6K and Erk1/2 was comparable between WT and Gαi2 SKO MEFs (Figure **1E**). Total Akt, S6K, S6 and Erk1/2 expression was again not significantly changed in Gαi1/Gαi2/Gαi3 SKO MEFs (Figure **1D** and **E**).

### Gαi1 and Gαi3 are required for Netrin-1-induced Akt-mTOR and Erk activation in Cas9-Gαi1/3 DKO” MEFs

To further support the role Gαi1 and Gαi3 in Netrin-1-activated signaling, the CRISPR (clustered, regularly interspaced, short palindromic repeats)/Cas9 (CRISPR-associated protein 9) gene editing method 21, 22, 28, 29 was employed to deplete Gαi1 and Gαi3 in WT MEFs. Following stable cell selection and Gαi1/3 KO verification, the “Cas9-Gαi1/3 DKO” MEFs 21, 22, 28, 29 were formed. As shown, treatment with Netrin-1 (25 ng/mL) increased phosphorylation of Akt, S6K and Erk1/2 in control MEFs with Cas9-C control vector (“Cas9-C”) (Figure **S1A**). In Cas9-Gαi1/3 DKO MEFs, Netrin-1-induced Akt-mTOR and Erk activation was completely blocked (Figure **S1A**). Total Akt, S6K and Erk1/2 expression was again unchanged (Figure **S1A**).

Next, Gαi1 shRNA-containing lentiviral particles and Gαi3 shRNA-containing lentiviral particles 21, 22, 28, 29 were co-added to WT MEFs. Following puromycin selection, stable MEFs were formed and were named as “shGαi1/3” MEFs 21, 22, 28, 29. In comparison to control MEFs expressing a scramble shRNA (“shC”), Netrin-1 (25 ng/mL)-induced phosphorylation of Akt and Erk1/2 was significantly decreased in shGαi1/3 MEFs (Figure **S1B**). Total Akt and Erk1/2 expression was unaffected (Figure **S1B**). Thus, Gαi1/3 silencing prevents Netrin-1-induced Akt-mTOR and Erk activation.

To examine the effects of overexpression, Ad-Gαi1 and Ad-Gαi3 were co-transduced into WT MEFs, and stable Gαi1/3-overexpressing MEFs formed 21, 22, 28, 29 (“OE-Gαi1/3” MEFs). We found that Netrin-1-induced Akt and Erk1/2 phosphorylation was augmented in OE-Gαi1/3 MEFs (Figure **S1C**). Total Akt and Erk1/2 expression was unchanged (Figure **S1C**). Thus, Gαi1 plus Gαi3 overexpression augmented Netrin-1-induced Akt-mTOR and Erk activation, further supporting the essential roles of the two Gαi proteins in Netrin-1 signaling.

### Gαi1 and Gαi3 associate with Netrin-1-stimualted CD146, required for CD146 internalization

**Figure 2:**
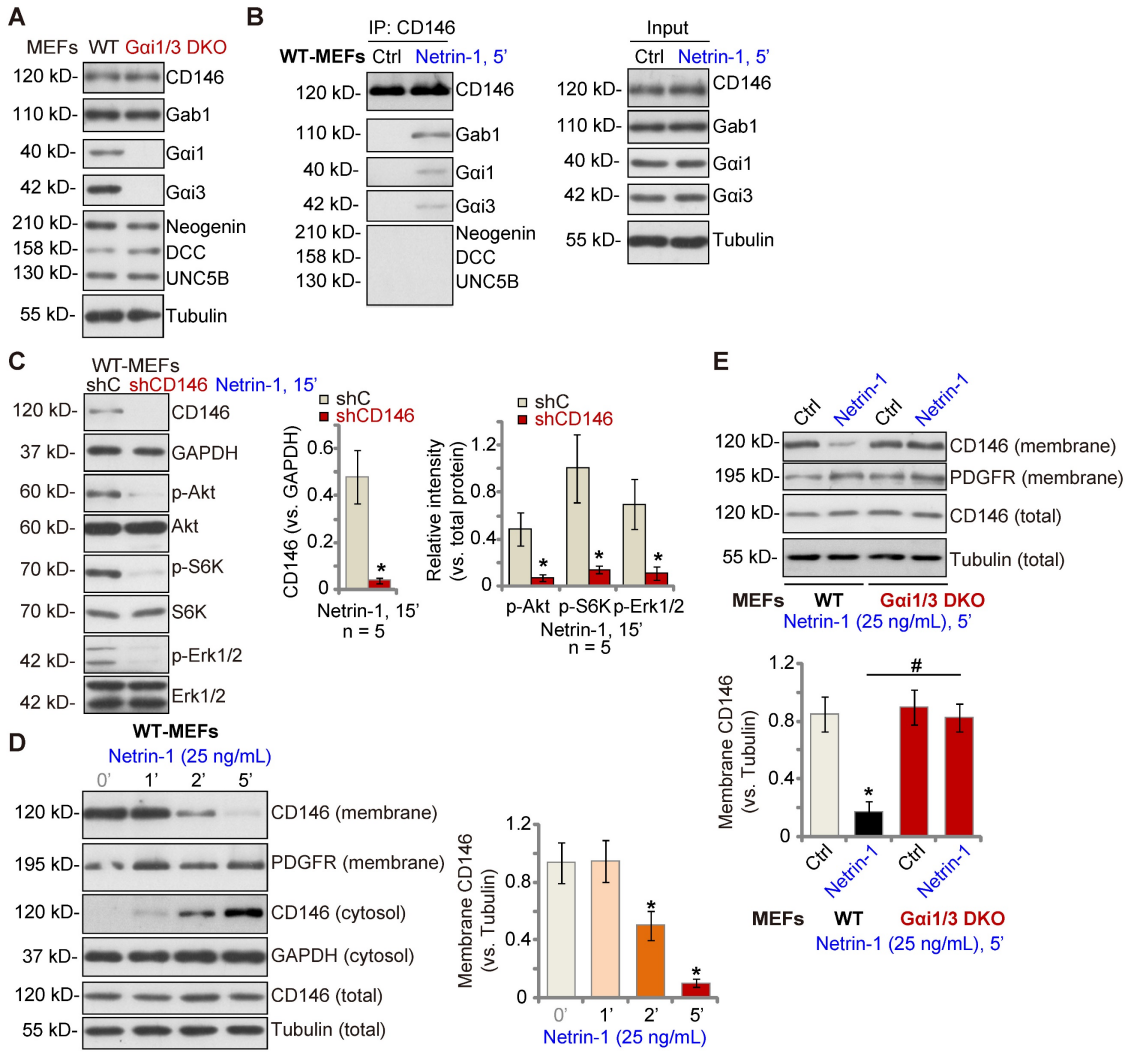
** Gαi1 and Gαi3 associate with Netrin-1-stimualted CD146, required for CD146 internalization.** Expression of listed signaling proteins of the Netrin-1 cascade in WT and Gαi1/3 DKO MEFs was shown (**A**). WT MEFs were treated with Netrin-1 (25 ng/mL) for 5 min, CD146-Gαi1/3-Gab1 association (“IP”) and expression (“Input”) was shown (**B**). DCC, Neogenin and UNC5B did not associate with Gαi1/3 in Netrin-1-treated WT MEFs (**B**). Puromycin-selected stable WT MEFs with CD146 shRNA (“shCD146”) or the scramble control shRNA (“shC”) were treated with Netrin-1 (25 ng/mL) for 15 min, expression of listed proteins was shown (**C**). WT MEFs were treated with Netrin-1 (25 ng/mL) for 1-5 min, expression of listed protein in membrane fraction lysates, cytosol fraction lysates and total cell lysates was tested (**D**). WT or Gαi1/3 DKO MEFs were treated with Netrin-1 (25 ng/mL) for 5 min, expression of listed protein in membrane fraction lysates and total cell lysates was tested (**E**). Quantifications were from five biological replicates (n = 5). *P < 0.05 versus “shC” or “0 min” (**D**). **^#^*P*** < 0.05.

Low concentrations of Netrin-1 bind CD146 to activate downstream signaling and angiogenesis 14, while Netrin-1 at high concentrations bind to DCC-UNC5B to suppress angiogenesis^14^. As Gαi1/3 DKO completely abolished Netrin-1-induced Akt-mTOR and Erk activation in MEFs, we examined whether Gαi1/3 DKO affected expression of key components of Netrin-1 signaling in MEFs. As shown, expression of DCC Netrin-1 receptor (DCC), Neogenin, UNC5B and CD146 was equivalent between WT and Gαi1/3 DKO MEFs (Figure **2A**). The co-immunoprecipitation (Co-IP) results showed that following Netrin-1 stimulation, Gαi1 and Gαi3 associated with CD146, but not UNC5B, DCC and Neogenin, in WT MEFs (Figure **2B**). Gab1, a key adaptor protein mediating Akt-mTOR and Erk activation 32-36, was also present in the CD146-Gαi1/3 complex in Netrin-1-treated MEFs (Figure **2B**). Significantly, shRNA-induced silencing of CD146 prevented Netrin-1-induced Akt-mTOR and Erk activation in MEFs (Figure **2C**).

Our previous studies have shown that Gαi1/3 associates with ligand-activated receptors to mediate receptor membrane internalization and downstream signaling 22, 23, 28, 37. Similarly, cell membrane-localized CD146 protein levels were rapidly decreased following treatment with Netrin-1 in WT MEFs (Figure **2D**), while cytosolic CD146 levels were significantly increased (Figure **2D**). Total CD146 protein and membrane-localized platelet-derived growth factor receptor (PGDFR) were unchanged (Figure **2D**). Importantly, CD146 internalization was prevented in Gαi1/3 DKO MEFs (Figure **2E**). Thus, Gαi1/3 association with Netrin-1-activated CD146 is required for CD146 internalization, essential for downstream signaling activation.

### The adaptor protein Gab1, downstream of Gαi1 and Gαi3, mediates Netrin-1-induced Akt-mTOR and Erk activation

Since the adaptor protein Gab1 was part of the CD146-Gαi1/3 complex in Netrin-1-treated MEFs (see Figure **2**), we tested its role in Netrin-1-induced signal transduction. In WT MEFs, following Netrin-1 treatment, Gab1 immunoprecipitated with CD146, Gαi1 and Gαi3 (Figure **S2A**), but did not associate with UNC5B, DCC and Neogenin (Figure **S2A**). Importantly, mimicking Gαi1/3 DKO's actions, Netrin-1-induced phosphorylation of Akt, S6K and Erk1/2 was completely abolished in Gab1 KO MEFs (Figure **S2B**), whereas Gαi1 and Gαi3 expression was intact (see our previous studies 22, 23, 28, 37). Gab1 acted as downstream of Gαi1 and Gαi3 as Netrin-1-induced Gab1 phosphorylation was completely abolished in Gαi1/3 DKO MEFs, but was partially attenuated in Gαi1 SKO MEFs or Gαi3 SKO MEFs (Figure **S2C**). Moreover, shRNA-induced silencing of Gαi1/3 (Figure **S2D**) or CRISPR/Cas9-induced Gαi1/3 DKO (Figure **S2E**) also prevented Netrin-1-induced Gab1 phosphorylation in MEFs. Ectopic overexpression of Gαi1 and Gαi3 further augmented Gab1 phosphorylation by Netrin-1 (Figure **S2F**). shRNA-induced silencing of CD146 also prevented Gab1 activation by Netrin-1 (Figure **S2G**).

### Gαi1/3 dominant negative mutants disrupt Netrin-1-induced CD146-Gαi1/3-Gab1 association, CD146 internalization and signaling activation

To further explore the underlying mechanisms of Gαi1/3-mediated Netrin-1 signaling, the previously-described dominant negative (DN) strategies were utilized 21-23, 29. The DN-Gαi1/3 constructs replaced the conserved Gly (G) residue with Thr (T) in G3 box, aiming to block Gαi1/3 association with adaptor/associated proteins 25, 26. The DN-Gαi1 and DN-Gαi3 constructs were co-transduced into WT MEFs, and after selection stable MEFs, “DN-Gαi1/3”, formed. We found that Netrin-1-induced CD146-Gab1-Gαi1/3 association was disrupted by DN-Gαi1/3 in WT MEFs (Figure **S3A**). Importantly, Netrin-1-induced CD146 internalization was inhibited by DN-Gαi1/3 (Figure **S3B**). These results further supported that CD146-Gαi1/3 association was required for Netrin-1-induced CD146 internalization. Moreover, Netrin-1-induced phosphorylation of Gab1, Akt and Erk1/2 was largely inhibited by DN-Gαi1/3 (Figure **S3C**).

### Gαi1/3 silencing prevents Netrin-1-induced signaling and pro-angiogenesis activity* in vitro*

**Figure 3:**
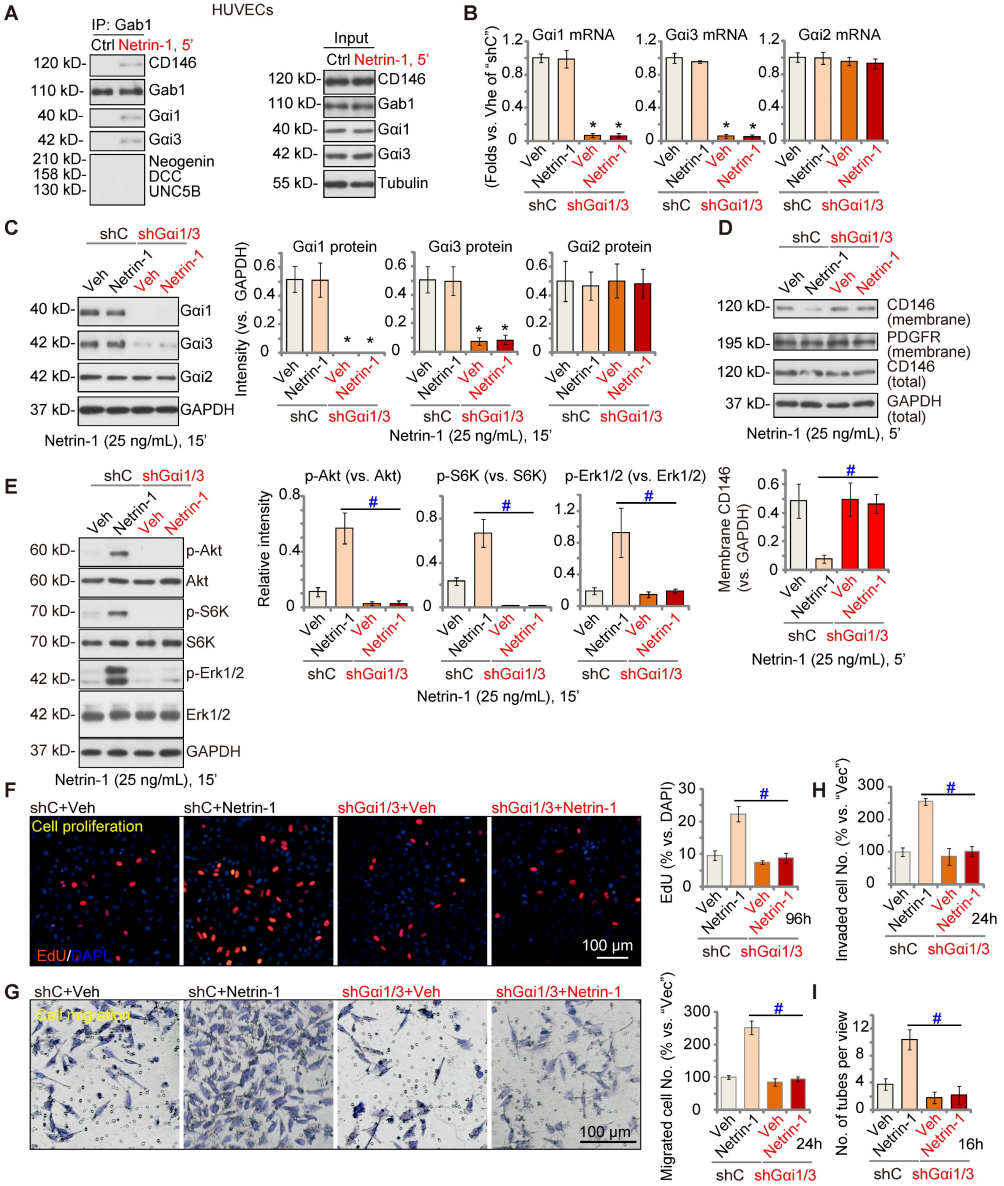
** Gαi1/3 shRNA prevents Netrin-1-induced signaling and pro-angiogenic activity in HUVECs.** HUVECs were treated with Netrin-1 (25 ng/mL) for 5 min, Gab1-immunoprecipitated proteins were tested by Co-IP assays (**A**), and expression of listed proteins was examined as well (“Input”) (**A**). Puromycin-selected stable HUVECs, with the lentiviral Gαi1 shRNA plus lentiviral Gαi3 shRNA (“shGαi1/3”) or scramble control shRNA (“shC”), were treated with or without Netrin-1 (25 ng/mL) for 15 min, expression of listed mRNAs and proteins was tested (**B**, **C** and **E**). The shGαi1/3 HUVECs or the shC HUVECs were treated with or without Netrin-1 (25 ng/mL) for 5 min, expression of listed protein in membrane fraction lysates and total cell lysates was tested (**D**); HUVECs were also cultivated for applied time periods, cell proliferation (by measuring EdU incorporation, **F**), migration (**G**), invasion (**H**) and tube formation (**I**) were tested by the described assays, with results quantified. The data were presented as mean ± standard deviation (SD, n = 5, five biological replicates). Quantifications were from five biological replicates (n = 5). * ***P*** < 0.05 versus “shC”.**^ #^*P*** < 0.05. Scale bar = 100 μm.

Next, we tested the potential role of Gαi1/3 in Netrin-1-induced signaling and pro-angiogenesis activity in endothelial cells. In cultured primary HUVECs, treatment with Netrin-1 (25 ng/mL) led to CD146-Gαi1/3-Gab1 association, and no association with DCC, Neogenin, or UNC5B (Figure **3A**). To silence Gαi1/3, Gαi1 and Gαi3 shRNA lentiviral particles 22, 29 were co-added to HUVECs, and following selection stable cells formed: “shGαi1/3” HUVECs. mRNA and protein expression of Gαi1 and Gαi3 were significantly decreased in shGαi1/3 HUVECs (Figure **3B**-**C**), whereas *Gαi2* mRNA and protein expression was unchanged (Figure **3B** and **C**). Significantly, Netrin-1 (25 ng/mL, 5')-induced CD146 internalization was abolished byGαi1/3 silencing in HUVECs (Figure **3D**), and Netrin-1-induced Akt, S6K and Erk1/2 phosphorylation was almost completely blocked (Figure **3E**). Total Akt, S6K and Erk1/2 expression was intact (Figure **3E**).

In shC control HUVECs, treatment with Netrin-1 promoted HUVEC proliferation and increased nuclear EdU incorporation (Figure **3F**), which was blocked byshGαi1/3 (Figure **3F**). Moreover, Netrin-1-induced *in vitro* cell migration (Figure **3G**), invasion (Figure **3H**) and tube formation (Figure **3I**) were prevented after Gαi1/3 silencing. In HUVEC, Netrin-1-induced cell proliferation (EdU incorporation, Figure **S4A**), migration (Figure **S4B**) and tube formation (Figure **S4C**) were significantly inhibited (but not blocked) by the Erk1/2 inhibitor PD98059 or the PI3K-Akt-mTOR inhibitor LY294002. Importantly, PD98059 plus LY294002 co-treatment completely blocked Netrin-1-induced pro-angiogenic actions in HUVECs (Figure **S4A**-**C**). These results supported that PI3K-Akt-mTOR and Erk are two key cascades required for Netrin-1-induced pro-angiogenic actions in endothelial cells.

### EctopicGαi1/3 overexpression amplifies Netrin-1-induced signaling and pro-angiogenesis activity *in vitro*

**Figure 4:**
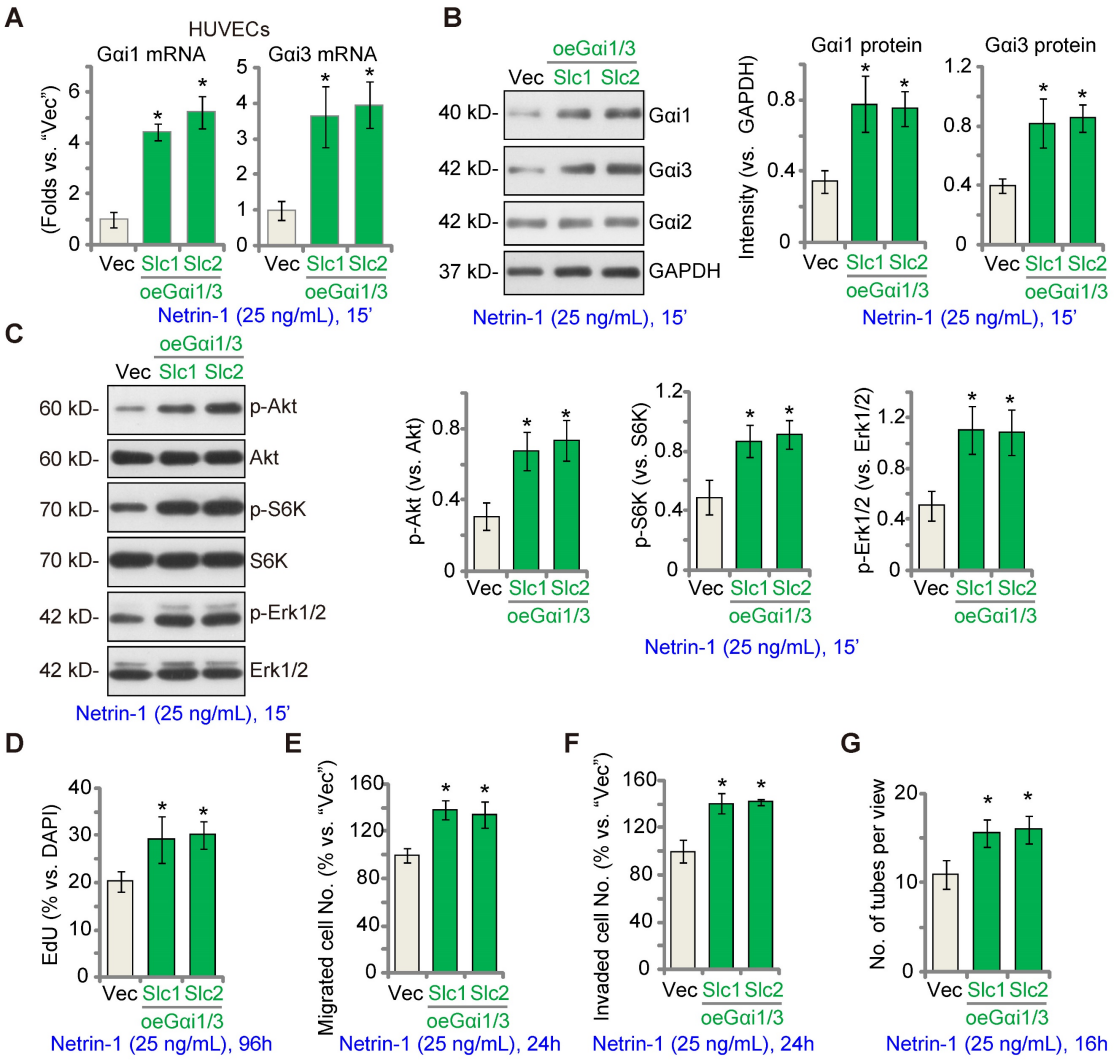
** Ectopic Gαi1/3 overexpression amplifies Netrin-1-induced signaling and pro-angiogenesis activity *in vitro.*** HUVECs were stably transduced the lentiviral Gαi1 expression construct plus the lentiviral Gαi3 expression construct, and puromycin was added to select two stable colonies, (“oeGαi1/3-Slc1” and “oeGαi1/3-Slc2”); Control HUVECs were transduced with the empty vector (“Vec”); Cells were treated with or without Netrin-1 (25 ng/mL) for 15 min, expression of listed mRNAs and proteins was tested (**A**-**C**). HUVECs were cultivated for applied time periods, cell proliferation (by measuring EdU-positive nuclei ratio, **D**), migration (**E**), invasion (**F**) and tube formation (**G**) were tested by the described assays, with results quantified. The data were presented as mean ± standard deviation (SD, n = 5, five biological replicates). Quantifications were from five biological replicates (n = 5). * ***P*** < 0.05 versus “Vec”.

We hypothesized that ectopic Gαi1/3 overexpression could augment Netrin-1-induced signaling and pro-angiogenesis activity. To test this hypothesis, Gαi1 and Gαi3 expressing lentiviral particles were co-added to HUVECs and puromycin was added to select two stable colonies, namely “oeGαi1/3-Slc1” and “oeGαi1/3-Slc2”. As compared to the vector control HUVECs (“Vec”), mRNA and protein expression of Gαi1 and Gαi3 was robustly increased in oeGαi1/3 HUVECs (Figure **4A**-**B**), whereas Gαi2 expression was unchanged (Figure 4**B**). Consequently, Akt, S6K and Erk1/2 phosphorylation in response to Netrin-1 was augmented in oeGαi1/3 HUVECs (Figure **4C**). Ectopic Gαi1/3 overexpression also amplified Netrin-1-induced pro-angiogenesis activity *in vitro*. In oeGαi1/3-Slc1 and oeGαi1/3-Slc2 HUVECs, Netrin-1-induced cell proliferation (EdU-positive nuclei ratio, Figure **4D**), *in vitro* cell migration (Figure **4E**), invasion (Figure **4F**) and tube formation (Figure **4G**) were significantly augmented.

### Endothelial knockdown of Gαi1/3 abolishes Netrin-1-induced retinal angiogenesis in mice

**Figure 5:**
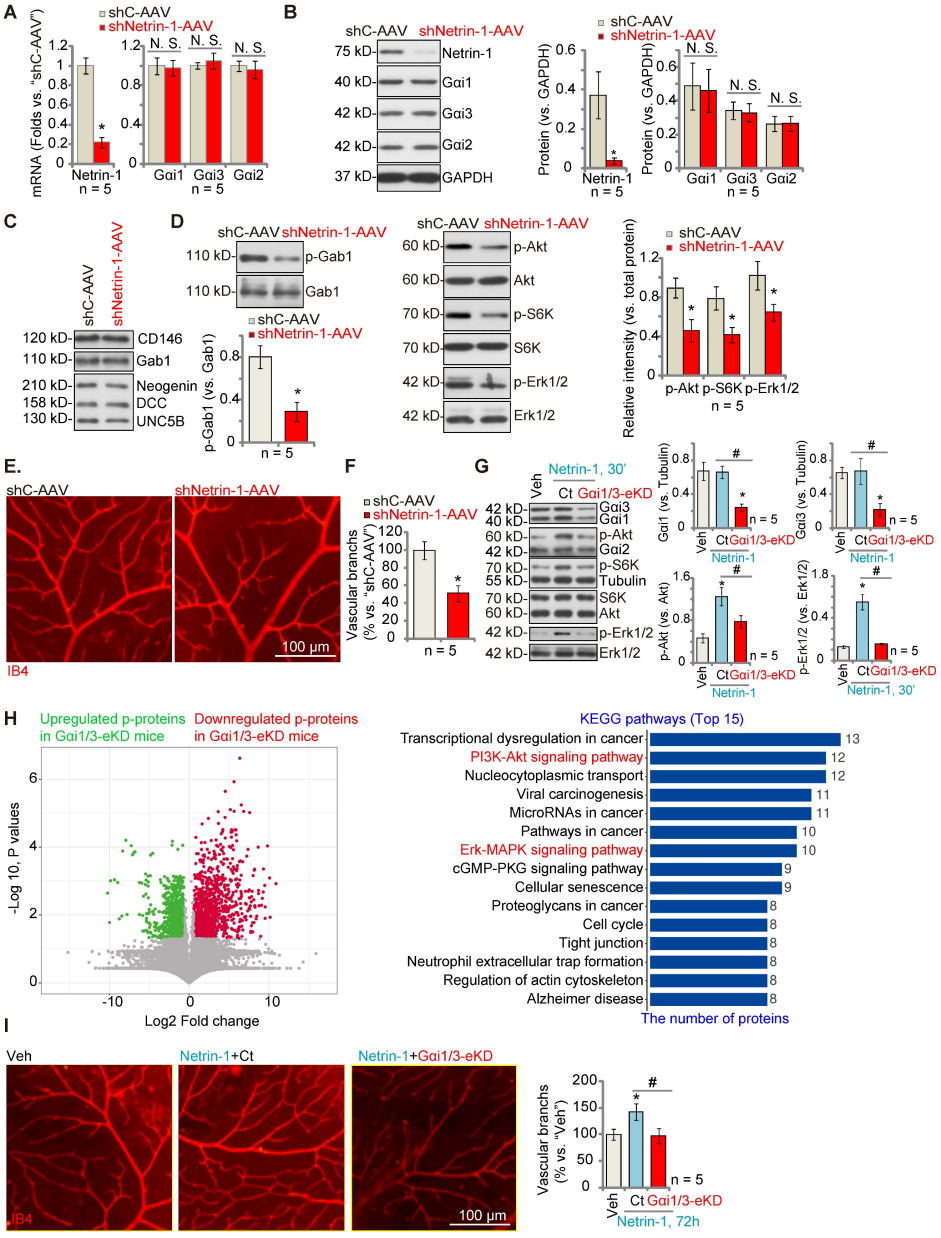
** Endothelial knockdown of Gαi1/3 abolishes Netrin-1-induced retinal angiogenesis in mice.** The C57B/6 adult mice were intravitreously injected with the AAV5 with Netrin-1 shRNA (“shNetrin-1-AAV”) or the AAV5 with scramble control shRNA (“shC-AAV”). After 10 days, expression of listed mRNAs and proteins in the retinal tissues was tested (**A-D** and **F**). The retinal vasculature was measured by IB4 staining (**E**) and the average number of vascular branches per view was calculated (**E**). The C57B/6 adult mice were intravitreously injected with the AAV5-TIE1-Gαi1 shRNA construct plus the AAV5-TIE1-Gαi3 shRNA construct (“Gαi1/3-eKD”). Control mice were intravitreously injected with the AAV5-TIE1-scramble control shRNA (“Ct”). After 10 days, mice were then intravitreously injected with Netrin-1 (0.25 ng in 0.1 μL); After 30 min, retinal tissues were collected and expression of listed proteins was shown (**G**); The retinal tissues of Netrin-1-challenged control mice and Gαi1/3-eKD mice were collected and subject to phosphoproteomics analyses. Differential phosphorylated proteins were shown in the volcano plot and KEGG pathway analyses were performed (**H**). Alternatively, the retinal vasculature was measured by IB4 staining after 72h (**I**), and the average number of vascular branches per view was calculated (**I**). The data were presented as mean ± standard deviation (SD, n = 5, five biological replicates). * ***P*** < 0.05 versus “shC-AAV” or vehicle control (“Veh”, saline). ^#^***P*** < 0.05. “N. S.” stands for non-statistical differences (***P*** > 0.05). Scale bar = 100 μm.

To investigate the role of Netrin-1 in angiogenesis *in vivo*, C57B/6 mice were intravitreously injected with AAV5 Netrin-1 shRNA (“shNetrin-1-AAV”). Control mice were injected with the AAV5 with a scramble shRNA (“shC-AAV”). Ten days after virus injection, the retinal tissues were homogenized and analyzed by qRT-PCR and Western blotting assays. As shown, mRNA and protein expression of Netrin-1 was significantly decreased in shNetrin-1-AAV-injected retinal tissues (Figure **5A**-**B**), whereas the mRNA and protein expression levels of Gαi1, Gαi2and Gαi3 were not altered (Figure **5A**-**B**). The protein expression of DCC, Neogenin, UNC5B, CD146 and Gab1 in retinal tissues was equivalent between shNetrin-1-AAV and shC-AAV mice (Figure **5C**). Significantly, phosphorylation of Gab1, Akt, S6K and Erk1/2 was decreased in shNetrin-1-AAV retinal tissues (Figure** 5D**). Endothelial staining with IB4, Figure 5**E**, revealed that shNetrin-1-AAV injection inhibited retinal angiogenesis *in vivo*. The retinas of shNetrin-1-AAV mice showed a significantly decreased number of vascular branches and branch points, and reduced retinal vascular complexity (Figure **5E**-**F**).

To explore the role of Gαi1/3 in Netrin-1-induced angiogenesis *in vivo*, C57B/6 mice were intravitreously injected with the AAV5-TIE1-Gαi1 shRNA plus the AAV5-TIE1-Gαi3 shRNA, leading to endothelial knockdown ofGαi1/3 (“Gαi1/3-eKD”, as reported previously 29). Expression of Gαi1 and Gαi3, but not Gαi2, was significantly decreased in retinal tissues of Gαi1/3-eKD mice (Figure **5G**). Importantly, intravitreous injection of Netrin-1 increased Akt, S6K and Erk1/2 phosphorylation in the retinal tissues of vector control (“Ct”) mice (Figure **5G**), which was largely inhibited in Gαi1/3-eKD mice (Figure **5G**).

The retinal tissues of Netrin-1-challenged control mice and Gαi1/3-eKD mice were collected and tissue lysates were subject to phosphoproteomics analyses. Differential phosphorylated proteins between the control and the Gαi1/3-eKD mice were shown in the volcano plot (Figure **5H**). These proteins were then subject to KEGG pathway analyses and top 15 pathways were shown (Figure **5H**). As demonstrated, besides the already-detected proteins in the PI3K-Akt-mTOR and Erk-MAPK cascades (Figure **5G**), the phosphorylation levels of the proteins in other signaling cascades also showed significant differences in Gαi1/3-eKD retina tissues (Figure **5H**). These pathways, including tight junction, transcriptional dysregulation in cancer, cancer-associated pathways, cell cycle, regulation of actin cytoskeleton and others (Figure **5H**), are also closely associated with the PI3K-Akt-mTOR/Erk-MAPK cascades and are important for angiogenesis 38-44. These results, together with the *in vitro* findings that PD98059 plus LY294002 co-treatment blocked Netrin-1-induced pro-angiogenic actions in HUVECs (Figure **S4**), supported that PI3K-Akt-mTOR and Erk-MAPK activation should be key downstream signaling effectors of Gαi1/3 in mediating Netrin-1-induced angiogenesis.

IB4 staining assay results, Figure **5I**, showed that Netrin-1 injection (for 48h) promoted retinal angiogenesis, causing an increased number of vascular branches and branch points, and enhanced retinal vascular complexity in control mice(Figure **5I**), whereas Netrin-1 effects were almost completely blocked in Gαi1/3-eKD mice (Figure **5I**). Thus, Gαi1/3 are important for Netrin-1-induced signaling and retinal angiogenesis *in vivo*.

### Silencing of Netrin-1 inhibits pathological retinal angiogenesis in diabetic retinopathy (DR) mice

**Figure 6:**
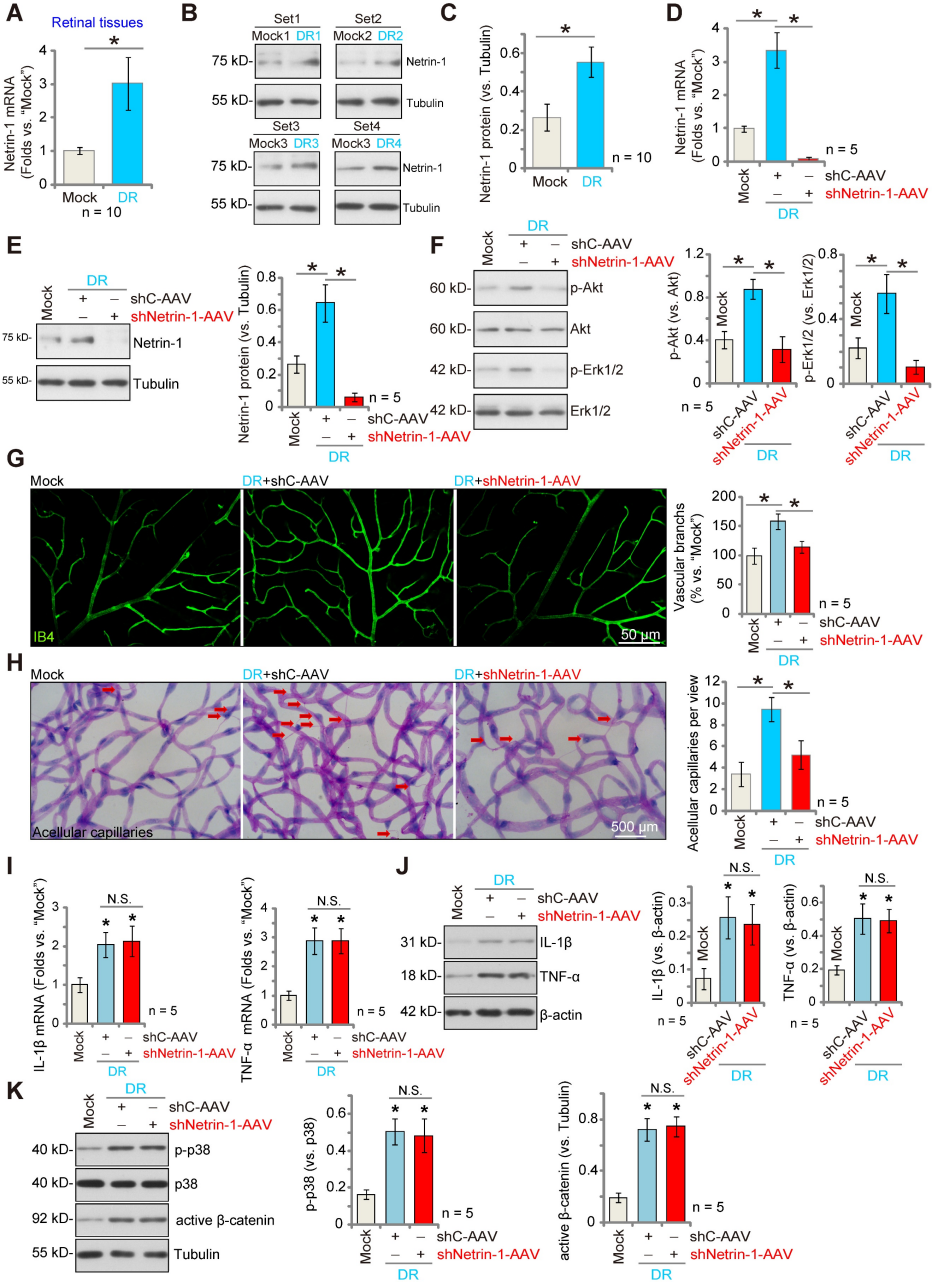
** Silence of Netrin-1 inhibits pathological retinal angiogenesis in diabetic retinopathy (DR) mice.** The retina tissues of STZ-administrated diabetic retinopathy mice (“DR”) and citrate buffer-administrated mock control mice (“Mock”) were separated, expression of *Netrin-1* mRNA and protein was tested, and results quantified (**A**-**C**). At day-30 following STZ administration, the DR mice were intravitreously injected with AAV5-packed Netrin-1 shRNA (“shNetrin-1-AAV”) or AAV5-packed scramble control shRNA (“shC-AAV”), each at 0.1 μL; At day-90 after last STZ administration, retinal tissues were separated and expression of listed mRNAs and proteins was tested (**D**-**F, I**-**K**). The retinal vasculature was measured by IB4 staining (**G**, scale bar = 50 μm), and the average number of vascular branches per view measured (**G**). The retinal trypsin digestion was performed to detect acellular capillaries and the number of acellular capillaries (red arrows) recorded (**H**, scale bar = 500 μm). The data were presented as mean ± standard deviation (SD, biological replicates).* ***P*** < 0.05. “N. S.” stands for non-statistical differences (***P*** > 0.05).

We examined whether Netrin-1 expression was altered in the retinas of DR mice. Three months after STZ administration, retinas from DR (STZ-administrated) and mock control (PBS-administrated) mice were collected. As shown, *Netrin-1* mRNA expression in retina tissues of DR mice was significantly higher than that in retinas of control mice (Figure **6A**). Moreover, Netrin-1 protein upregulation was detected in the retinas of four representative DR mice (Figure **6B**). When combining all 10 sets of the Netrin-1 blotting data, we found that Netrin-1 protein was significantly upregulated in retinas of DR mice (Figure **6C**).

To investigate the potential role of Netrin-1 in pathological angiogenesis in DR mice, AAV5-Netrin-1 shRNA (shNetrin-1-AAV) or AAV5-scramble control shRNA (“shC-AAV”) were intravitreously injected into retinas of DR mice on day-30 after the last STZ administration, and signaling proteins tested two months later. Western blotting assay results showed that shNetrin-1-AAV silenced *Netrin-1* mRNA and protein expression in retinal tissues of the DR mice (Figure **6D** and **E**). Netrin-1 was upregulated, and Akt and Erk1/2 phosphorylation increased in DR mice's retinas (Figure **6F**). Importantly, silencing of Netrin-1 inhibited Akt and Erk activation in the retinal tissues of DR mice (Figure **6F**).

In addition, IB4 staining supported pathological retinal angiogenesis in DR mice with shC-AAV injection, showing an increased number of vascular branches and branch points, and enhanced retinal vascular complexity (Figure **6G**). Moreover, the retinal trypsin digestion assay demonstrated that the number of retinal acellular capillaries were significantly increased in shC-AAV DR mice (Figure **6H**). Remarkably, decreasing Netrin-1 levels, by intravitreous injection of shNetrin-1-AAV, ameliorated the pathological retinal angiogenesis in DR mice. Retinal pathological angiogenesis (IB4 staining, Figure **6G**) and acellular capillary formation (Figure **6H**) in the DR mice were largely inhibited after injection of shNetrin-1-AAV. These results support that decreasing Netrin-1 expression inhibited pathological retinal angiogenesis in DR mice.

Notably, mRNA and protein expression of inflammatory cytokines, including IL-1β and tumor necrosis factor-α (TNF-α), were significantly increased in the retinal tissues of DR mice, indicative of inflammation (Figure **6I**-**J**). Moreover, p38 phosphorylation and the active β-catenin levels were also increased (Figure **6K**). Netrin-1 silencing failed to affect inflammation as well as p38 and β-catenin activation in the retinal tissues of DR mice (Figure **6I**-**K**).

### Silencing of Netrin-1 inhibits retinal ganglion cells (RGCs) degeneration in diabetic retinopathy (DR) mice

**Figure 7:**
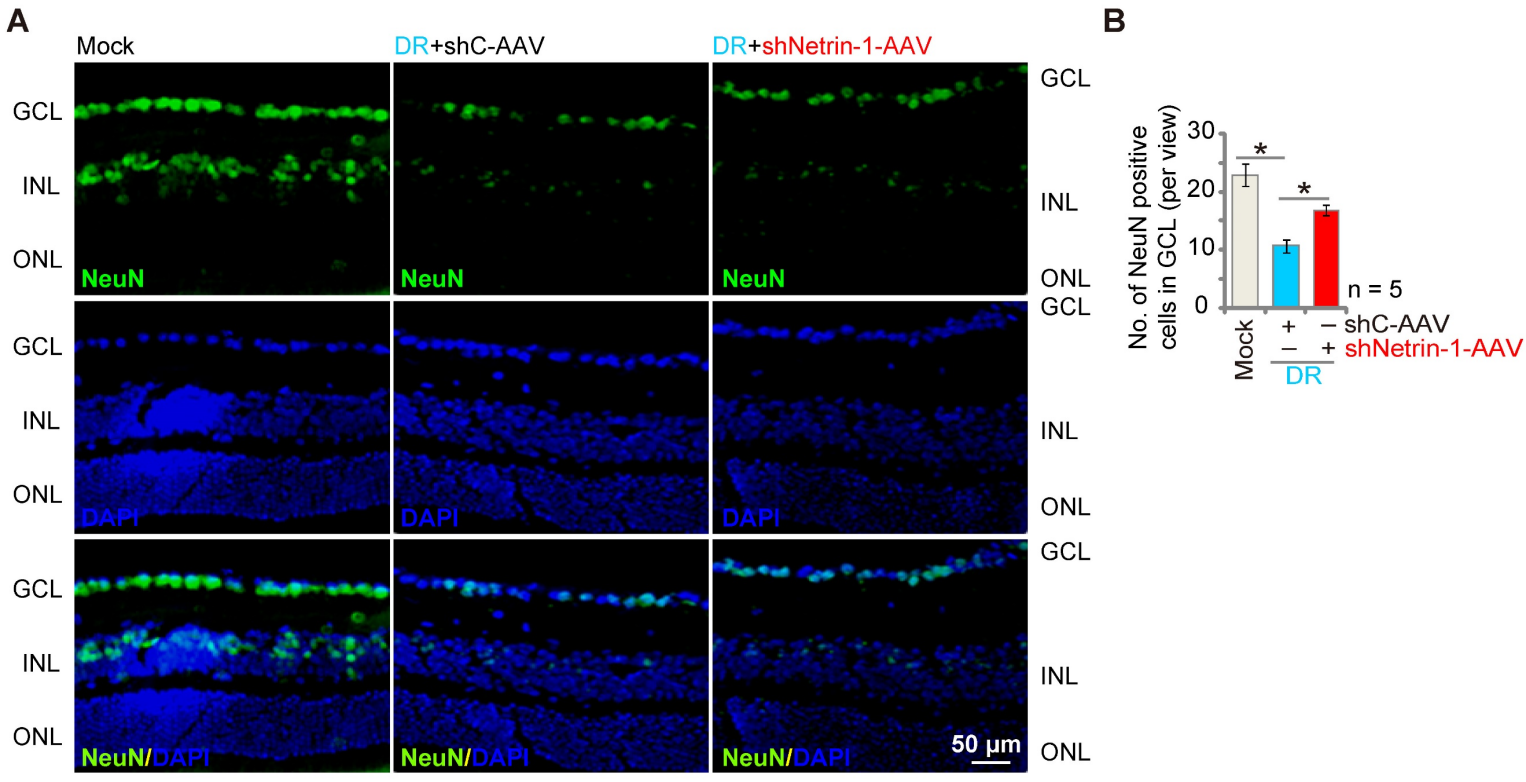
** Silence of Netrin-1 inhibits RGC degeneration in diabetic retinopathy (DR) mice.** At day-30 following last STZ administration, the DR mice were intravitreously injected with AAV5-packed Netrin-1 shRNA (“shNetrin-1-AAV”) or AAV5-packed scramble control shRNA (“shC-AAV”), each at 0.1 μL; At day-90 after STZ administration. NeuN immunofluorescence staining in the retinal slides of the mice were shown, and the number of NeuN-positive RGCs in GCL recorded (**A** and **B**).The data were presented as mean ± standard deviation (SD, biological replicates). “Mock” stands for mice with citrate buffer administration. “GCL”: ganglion cell layer, “ONL”: Outer nuclear layer, “INL”: Inner nuclear layer. * ***P*** < 0.05. Scale bar = 50 μm.

DR mice display significant neuronal degeneration possibly due to pathological angiogenesis, disrupted energy supply, inflammation and oxidative stress 45, 46. Staining with the neuronal marker NeuN, we found that the number of NeuN-stained RGCs was substantially decreased in retinas of shC-AAV DR mice (Figure **7A**-**B**). Importantly, shNetrin-1-AAV-induced knockdown of Netrin-1 ameliorated RGCs degeneration in DR mice (Figure** 7A**-**B**).

### Netrin-1-CD146 expression is increased in the proliferative retinal tissues of human PDR patients

**Figure 8:**
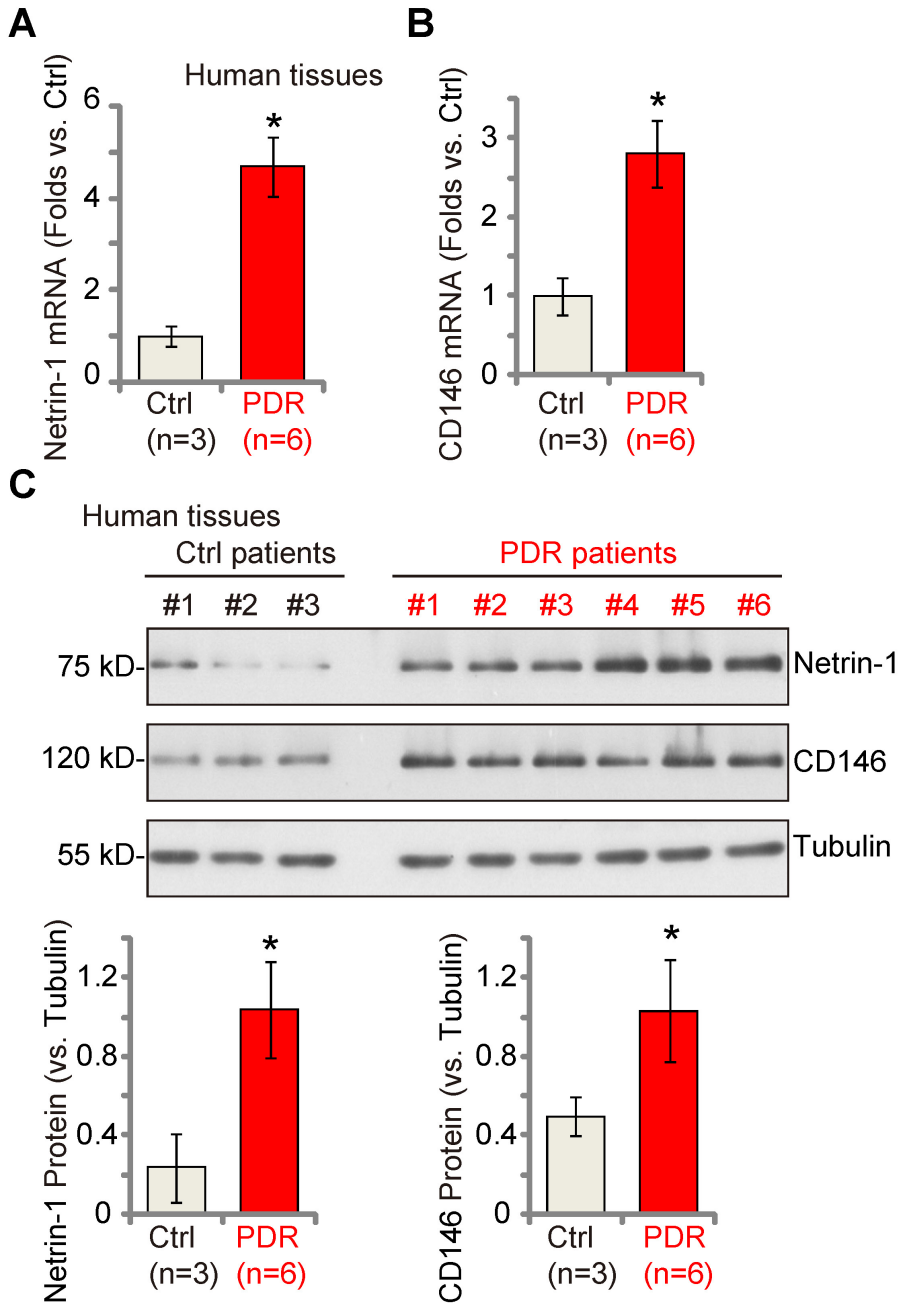
** Netrin-1 and CD146 expression is increased in the proliferative retinal tissues of human proliferative diabetic retinopathy (PDR) patients.** The proliferative retinal membrane of six different PDR patients and retinas of three age-matched traumatic retinectomy patients (“Ctrl”) were homogenized, and mRNA and protein expression of* Netrin-1* and CD146 in the fresh tissue lysates was examined (**A**-**C**). The data were presented as mean ± standard deviation (SD, biological replicates). * ***P*** < 0.05 versus “Ctrl”.

Lastly, we examined whether Netrin-1 and CD146 expression was dysregulated in the proliferative retinal tissues of human patients. As described previously 18, 22, the retinal proliferative membrane tissues of six different human PDR patients, as well as retinas of three age-matched traumatic retinectomy patients were investigated. Fresh tissue lysates were examined. We found that *Netrin-1* and *CD146* mRNA (Figure **8A**-**B**) and protein (Figure **8C**) expression was significantly increased in the proliferative retinal tissues of human PDR patients.

## Discussion

Dependent on the doses utilized, Netrin-1 can either inhibit or promote angiogenesis 10-12, 14, 47-50. Xu *et al.,* have shown that Netrin-1 expression was significantly increased in retinal tissues of oxygen-induced retinopathy (OIR) mice 51, and decreasing Netrin-1 suppressed retinal neovascularization in OIR mice 51. Han *et al.,* however found that the expression of Netrin-1 and its receptor UNC5B was decreased in rat corneal epithelium after corneal alkali burn 52. Interestingly, exogenous administration of Netrin-1 onto rat ocular surfaces was shown to suppress corneal neovascularization 52. Here we found that *Netrin-1* mRNA and protein expression was significantly increased in the retinal tissues of DR mice. Importantly, Netrin-1 silencing by intravitreous injection of shNetrin-1 AAV largely inhibited pathological retinal angiogenesis in DR mice. Contrarily, intravitreous injection of Netrin-1 increased retinal angiogenesis in mice. These results support that Netrin-1 is a pro-angiogenic factor in mouse retina.

Low concentrations of Netrin-1 bind to the high-affinity receptor CD146 to promote angiogenesis 14. The underlying mechanisms of Netrin-1-CD146-activated angiogenesis are still elusive. Here, we show that Gαi1 and Gαi3 are essential proteins in mediating Netrin-1-induced signaling. In MEFs, Gαi1 and Gαi3 KO (using DKO/SKO MEFs or the CRISPR/Cas9 strategy), silencing (by shRNA) or dominant negative mutations largely inhibited Netrin-1-induced Akt-mTOR and Erk activation. In contrast, Gαi1 and Gαi3 overexpression augmented Netrin-1-induced signaling. In HUVECs Netrin-1-induced Akt-mTOR and Erk activation was prevented by Gαi1 and Gαi3 shRNA, but amplified after Gαi1 and Gαi3 overexpression.

Our studies have demonstrated a pivotal role of Gαi1 and Gαi3 in angiogenesis. Gαi1/3 silencing by shRNA lentivirus suppressed alkali burn-induced neovascularization in mouse cornea and decreased OIR-induced retinal neovascularization 22. Moreover, alkali burn- and OIR-induced angiogenesis was robustly inhibited in Gαi1/3 double knockout mice 22. PCK1 increased Gαi3 expression and downstream Akt-mTOR activation, promoting endothelial cell proliferation, migration, sprouting, and tube formation *in vitro* and retinal angiogenesis *in vivo* 18. Moreover, R-spondin3 induced leucine rich repeat containing G protein-coupled receptor 4 (LGR4)-Gαi1/3-Gab1 complex formation, required for Akt-mTOR activation, and decreasing Gαi1/3 expression inhibited R-spondin3-induced angiogenesis 29.

In the present study, we found that Gαi1 and Gαi3 are essential signaling proteins mediating Netrin-1-induced angiogenesis. Netrin-1-induced HUVEC proliferation, *in vitro* migration, invasion and tube formation were largely inhibited by Gαi1/3 double silencing, but were augmented after Gαi1 and Gαi3 overexpression. Importantly, endothelial knockdown of Gαi1/3 significantly inhibited Netrin1-induced signaling and retinal angiogenesis in mice.

CD146 expression in the endothelial cells is vital for cell proliferation, migration and tube formation *in vitro* 53, 54 and angiogenesis *in vivo* 55-58. Moreover, CD146 can also act as a co-receptor for VEGFR2, promoting VEGF signaling in endothelial cells 59. Silencing or block (using a monoclonal antibody) of CD146 inhibited angiogenesis 58, 60.We have previously shown that Gαi1 and Gαi3 association with receptors is required for receptor internalization 22, 23, 28, 37. With VEGF stimulation, Gαi1/3 dictated VEGFR2 endocytosis and subsequent signal transduction 22. Upon IL-4 treatment, Gαi1/3 immunoprecipitated with the intracellular domain of IL-4Rαin macrophages, mediating IL-4Rαendosomal trafficking and downstream Gab1-Akt activation 28. LPS stimulation resulted in Gαi1/3 association with CD14 in macrophages, promoting TLR4 endocytosis 37. Similarly, Gαi1/3 silencing or mutation in neurons inhibited BDNF-induced TrkB endocytosis and activation of downstream signaling 23.

In the present study, we show that Netrin-1 induced Gαi1/3 association with CD146 is required for CD146 internalization and Gab1 recruitment. Gαi1/3 KO, silencing or dominant negative mutations inhibited Netrin-1-induced CD146 internalization and Gab1 activation in MEFs and HUVECs. Therefore, Gαi1/3-mediated CD146 membrane internalization maybe a key mechanism for Netrin-1-activated signaling and angiogenesis.

Previous studies have proposed that Netrin-1 could act as a pro-angiogenesis factor and promote retinopathy progression in DR 12, 15, 47. Netrin-1 is able to attract both endothelial cells and axons simultaneously 48-50, and injection of low concentrations of Netrin-1 (100 ng/mL) accelerated retinal neovascularization in STZ-induced diabetic rats 15. We found that *Netrin-1* mRNA and protein expression as well as Akt-Erk activation are significantly elevated in retinal tissues of DR mice. Decreasing Netrin-1 levels, by intravitreous Netrin-1 shRNA AAV injection, inhibited Akt-Erk activation, pathological retinal angiogenesis and RGC degeneration in DR mice. Importantly, mRNA and protein expression of Netrin-1 and CD146 was significantly elevated in the proliferative retinal tissues of PDR patients. These results, together with the previous findings showing Gαi1/3 upregulation in proliferative retinal tissues of PDR patients 22, further implicates increased Netrin-1-CD146-Gαi1/3 signaling in abnormal angiogenesis of PDR.

## Conclusion

Netrin-1 induces CD146-Gαi1/3-Gab1 complex formation to mediate downstream Akt-mTOR and Erk activation, important for angiogenesis *in vitro* and *in vivo*. DR is recognized as a microvascular disease. The anti-VEGF therapies have displayed remarkable efficacy in certain DR patients 61-63; Yet many patients are still unable to achieve clinically-significant visual improvement 61-63. As many other growth factors and various inflammatory cytokines also participate in the pathological angiogenesis in DR 61-63, it is extremely important to identify novel therapeutic targets required for DR progression 61-63. Our results imply that Netrin-1-CD146-Gαi1/3 signaling is responsible for abnormal angiogenesis of DR. This cascade represents a novel molecular therapeutic target of DR.

## Supplementary Material

Supplementary figures.

## Figures and Tables

**Table 1:** Antibodies utilized in the present study.

Antibodies	Company	Catalog Number	Concentration
Gαi1	Santa Cruz Biotechnology	sc-515658	1:2000
Gαi2	Santa Cruz Biotechnology	sc-13534	1:2000
Gαi3	Santa Cruz Biotechnology	sc-365422	1:2000
p-Akt S473	Cell Signaling Technology	9271	1:2000
Akt	Santa Cruz Biotechnology	sc-56878	1:5000
p-S6K	Cell Signaling Technology	9234	1:2000
S6K	Santa Cruz Biotechnology	sc-8418	1:2000
p-S6	Cell Signaling Technology	4856	1:5000
S6	Santa Cruz Biotechnology	sc-74576	1:10000
p-Erk1/2	Cell Signaling Technology	9101	1:5000
Erk1/2	Santa Cruz Biotechnology	sc-514302	1:5000
GAPDH	Proteintech	60004-1-Ig	1:10000
p-Gab1	Cell Signaling Technology	3233	1:2000
Gab1	Santa Cruz Biotechnology	sc-133191	1:2000
Neogenin	Cell Signaling Technology	39447	1:2000
UNC5B	Cell Signaling Technology	13851	1:2000
DCC	Abcam	ab273570	1:2000
CD146	Cell Signaling Technology	68706	1:2000
Tubulin	Proteintech	66031-1-Ig	1:10000
PDGFR	Cell Signaling Technology	3164	1:2000
Netrin-1	Abcam	ab126729	1:1000
p-p38	Cell Signaling Technology	9211	1:2000
p38	Cell Signaling Technology	8690	1:5000
Active β-Catenin	Cell Signaling Technology	19807	1:1000

## Abbreviations

EdU: 5-ethynyl-2'-deoxyuridine
BDNF: brain-derived neurotrophic factor
cAMP: cyclic AMP
CRISPR: clustered, regularly interspaced, short palindromic repeats
Cas9: CRISPR-associated protein 9
Co-IP: co-immunoprecipitation
DR: diabetic retinopathy
DCC: DCC Netrin-1 receptor
DN: dominant negative
DKO: double knockout
EGF: epidermal growth factor
FBS: fetal bovine serum
Gαi: G protein subunit alpha I
GCL: ganglion cell layer
Gab1: Grb2 associated binding protein 1
HUVEC: human umbilical vein endothelial cell
LGR4: leucine rich repeat containing G protein-coupled receptor 4
MEFs: mouse embryonic fibroblasts
IL-4: interleukin-4
IB4: isolectin B4
KGF: keratinocyte growth factor
mTOR: mammalian target of rapamycin
OIR: oxygen-induced retinopathy
PCK1: phosphoenolpyruvate carboxykinase 1
PDR: proliferative diabetic retinopathy
qRT-PCR: quantitative real-time PCR
S6K: ribosomal protein S6 kinase
RTKs: receptor tyrosine kinases
RGCs: retinal ganglion cells
shRNA: short hairpin RNA
SKO: single knockout
SD: standard deviation
STZ: streptozotocin
TNF-α: tumor necrosis factor-α
UNC5B: Unc-5 Netrin receptor B
VCAM-1: vascular cell adhesion molecule-1
VEGF: vascular endothelial growth factor
vWF: von willebrand factor
WT: wild-type
