# Supplementary material for: Gαi1/3 mediate Netrin-1-CD146-activated signaling and angiogenesis
Source: Theranostics. 2023 Apr 17;13(7):2319–36. doi: 10.7150/thno.80749 (PMC10157725; doi:10.7150/thno.80749)
Supplement: Supplementary file 1 — Supplementary figures. [file thnov13p2319s1.pdf]

Figure S1.

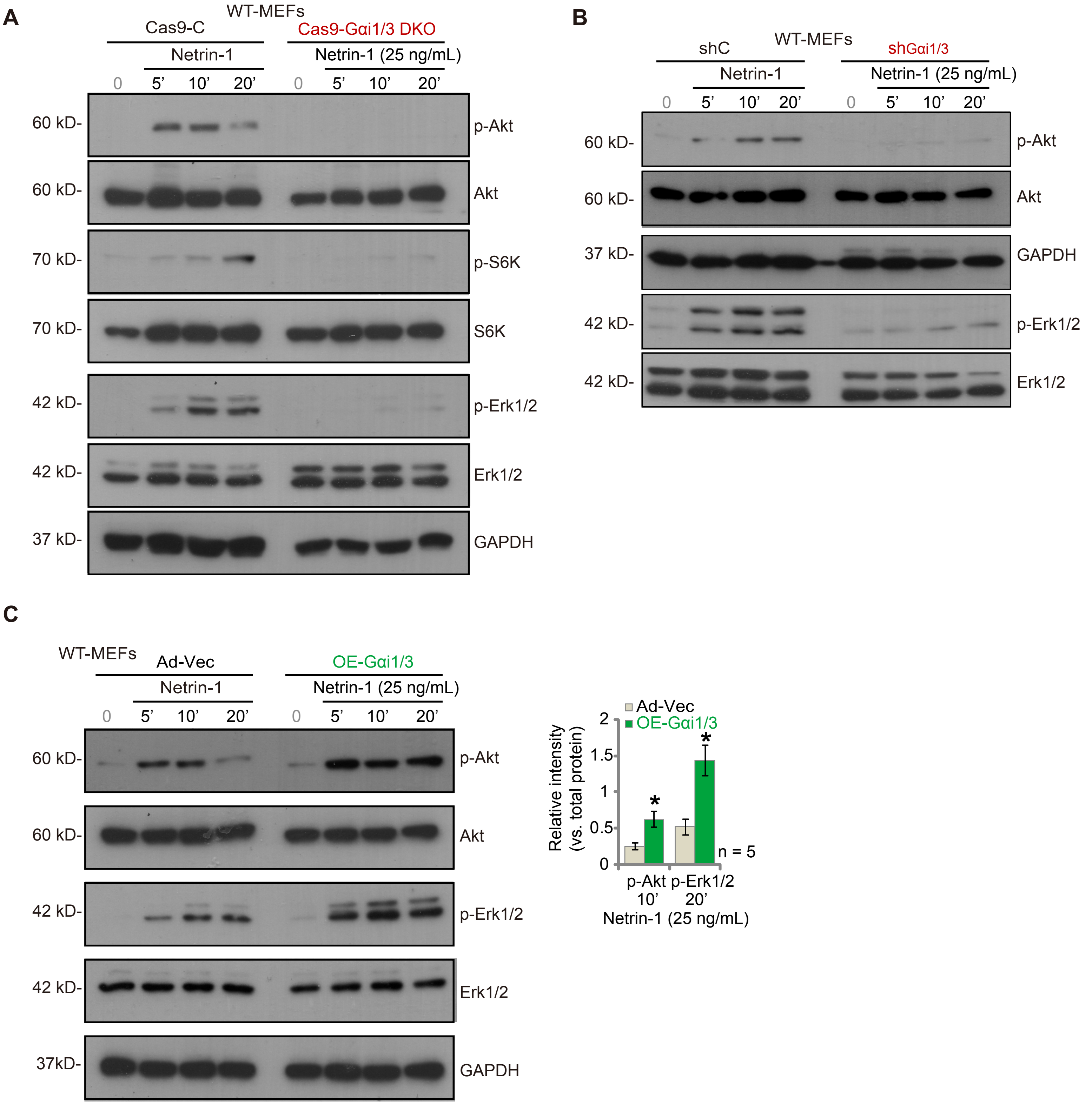
A
WT-MEFs
Cas9-C
Cas9-Gαi1/3 DKO
Netrin-1
Netrin-1 (25 ng/mL)
0
5’
10’
20’
0
5’
10’
20’
60 kD-
p-Akt
60 kD-
Akt
70 kD-
p-S6K
70 kD-
S6K
42 kD-
p-Erk1/2
42 kD-
Erk1/2
37 kD-
GAPDH
B
WT-MEFs
shC
shGαi1/3
Netrin-1
Netrin-1 (25 ng/mL)
0
5’
10’
20’
0
5’
10’
20’
60 kD-
p-Akt
60 kD-
Akt
37 kD-
GAPDH
42 kD-
p-Erk1/2
42 kD-
Erk1/2
C
WT-MEFs
Ad-Vec
OE-Gαi1/3
Netrin-1
Netrin-1 (25 ng/mL)
0
5’
10’
20’
0
5’
10’
20’
60 kD-
p-Akt
60 kD-
Akt
42 kD-
p-Erk1/2
42 kD-
Erk1/2
37kD-
GAPDH
Ad-Vec
OE-Gαi1/3
Relative intensity (vs. total protein)
2
1.5
1
0.5
0
*
*
n = 5
p-Akt 10’
p-Erk1/2 20’
Netrin-1 (25 ng/mL)

Figure S1. Gαi1 and Gαi3 are required for Netrin-1-induced Akt-mTOR and Erk activation in MEFs. Puromycin-selected stable WT MEFs with the CRISPR-Gαi1-KO construct plus the CRISPR-Gαi3-KO construct ("Cas9-Gαi1/3-DKO"), the Cas9 control construct ("Cas9-C"), the lentiviral Gαi1 shRNA plus the lentiviral Gαi3 shRNA ("shGαi1/3") or the scramble control shRNA ("shC") were cultivated and treated with Netrin-1 (25 ng/mL) for applied time periods, expression of listed proteins was shown, and protein phosphorylation was quantified (**A** and **B**). WT MEFs were stably transduced with the murine Gαi1 expression construct, the murine Gαi3 expression construct ("OE-Gαi1/3") or the empty vector ("Ad-Vec"), and were treated with Netrin-1 (25 ng/mL) and cultured for indicated time periods, expression of listed proteins was shown (**C**), and protein phosphorylation was quantified (**C**). Data were expressed as mean ± standard deviation (SD, biological replicates). Quantifications were from five biological replicates (n = 5). * $P < 0.05$ versus "Ad-Vec". $^{\#}P < 0.05$.

Figure S2

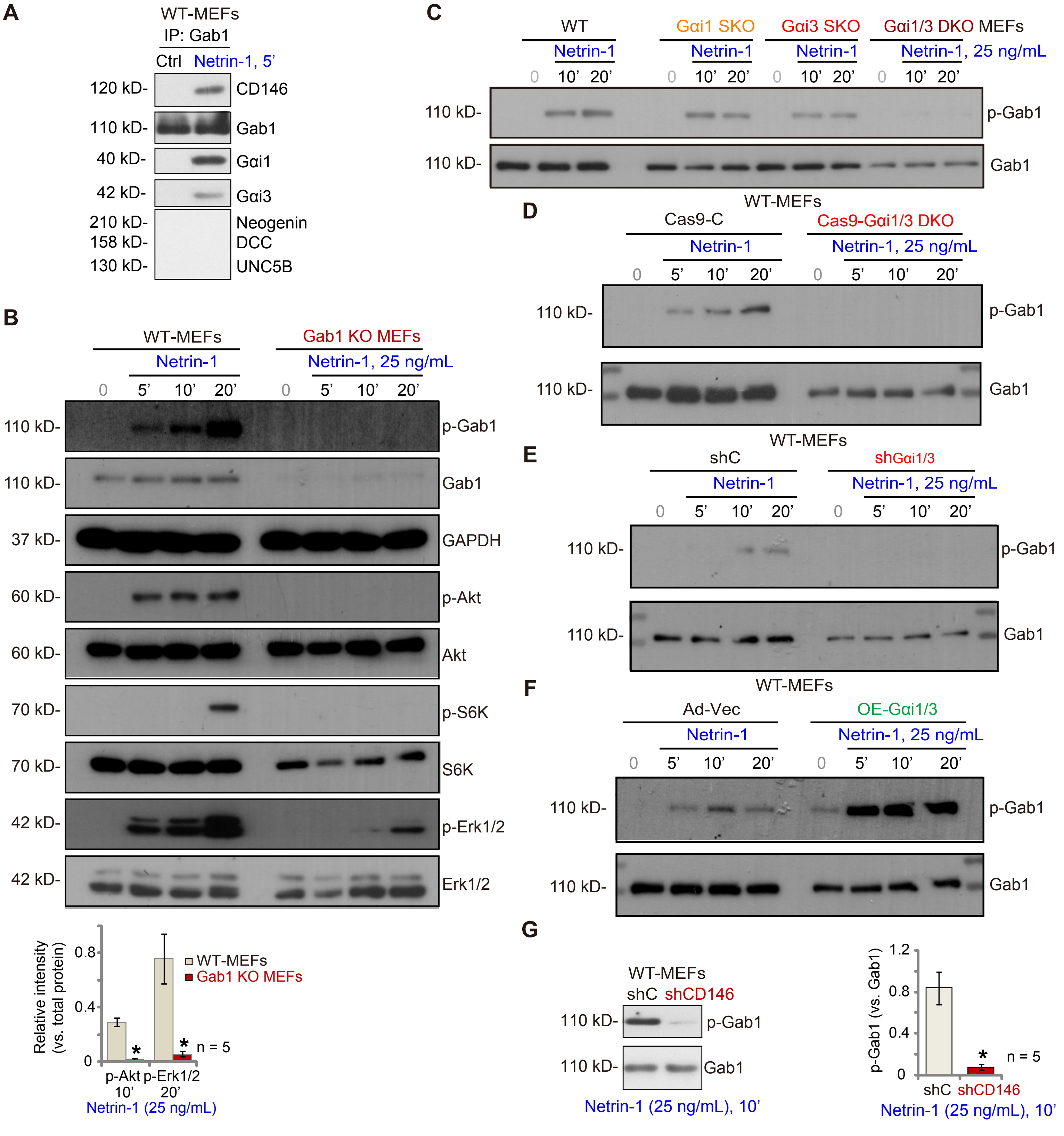
A
WT-MEFs
IP: Gab1
Ctrl
Netrin-1, 5'
120 kD-
110 kD-
40 kD-
42 kD-
210 kD-
158 kD-
130 kD-
CD146
Gab1
Gαi1
Gαi3
Neogenin
DCC
UNC5B
B
WT-MEFs
Gab1 KO MEFs
Netrin-1
Netrin-1, 25 ng/mL
0 5' 10' 20' 0 5' 10' 20'
110 kD- p-Gab1
110 kD- Gab1
37 kD- GAPDH
60 kD- p-Akt
60 kD- Akt
70 kD- p-S6K
70 kD- S6K
42 kD- p-Erk1/2
42 kD- Erk1/2
Relative intensity (vs. total protein)
0.8
0.4
0
WT-MEFs
Gab1 KO MEFs
n = 5
p-Akt 10'
p-Erk1/2 20'
Netrin-1 (25 ng/mL)
C
WT
Gαi1 SKO
Gαi3 SKO
Gαi1/3 DKO MEFs
Netrin-1
Netrin-1, 25 ng/mL
0 10' 20'
110 kD- p-Gab1
110 kD- Gab1
D
WT-MEFs
Cas9-C
Cas9-Gαi1/3 DKO
Netrin-1
Netrin-1, 25 ng/mL
0 5' 10' 20' 0 5' 10' 20'
110 kD- p-Gab1
110 kD- Gab1
E
WT-MEFs
shC
shGαi1/3
Netrin-1
Netrin-1, 25 ng/mL
0 5' 10' 20' 0 5' 10' 20'
110 kD- p-Gab1
110 kD- Gab1
F
WT-MEFs
Ad-Vec
OE-Gαi1/3
Netrin-1
Netrin-1, 25 ng/mL
0 5' 10' 20' 0 5' 10' 20'
110 kD- p-Gab1
110 kD- Gab1
G
WT-MEFs
shC shCD146
110 kD- p-Gab1
110 kD- Gab1
Netrin-1 (25 ng/mL), 10'
p-Gab1 (vs. Gab1)
1.2
0.8
0.4
0
n = 5
shC
shCD146
Netrin-1 (25 ng/mL), 10'

Figure S2. The adaptor protein Gab1, downstream of Gαi1 and Gαi3, mediates Netrin-1-induced Akt-mTOR and Erk activation. WT MEFs were treated with Netrin-1 (25 ng/mL) for 5 min, Gab1-immunopercipaited proteins was tested by Co-IP assays (**A**, "IP: Gab1"). WT and Gab1 KO MEFs were treated with Netrin-1 (25 ng/mL) for designated time, expression of listed proteins was shown, and protein phosphorylation was quantified (**B**). WT MEFs, Gαi1 or Gαi3 single knockout (SKO) MEFs, Gαi1/3 DKO MEFs, or WT MEFs with the CRISPR-Gαi1-KO construct plus the CRISPR-Gαi3-KO construct ("Cas9-Gαi1/3-DKO"), the Cas9 control construct ("Cas9-C"), the Gαi1 shRNA plus the Gαi3 shRNA ("shGαi1/3") or the scramble control shRNA ("shC"), the adenoviral mouse Gαi1 expression construct plus the adenoviral mouse Gαi3 expression construct ("OE-Gαi1/3") or the empty vector ("Ad-Vec"), CD146 shRNA ("shCD146") or "shC", were cultivated and treated Netrin-1 (25 ng/mL) for designated time, expression of listed proteins was shown (**C**-**G**), and protein phosphorylation was quantified (**G**). Quantifications were from five biological replicates (n = 5). *$\boldsymbol{P} < 0.05$ versus "WT" MEFs or "shC" treatment.

Figure S3

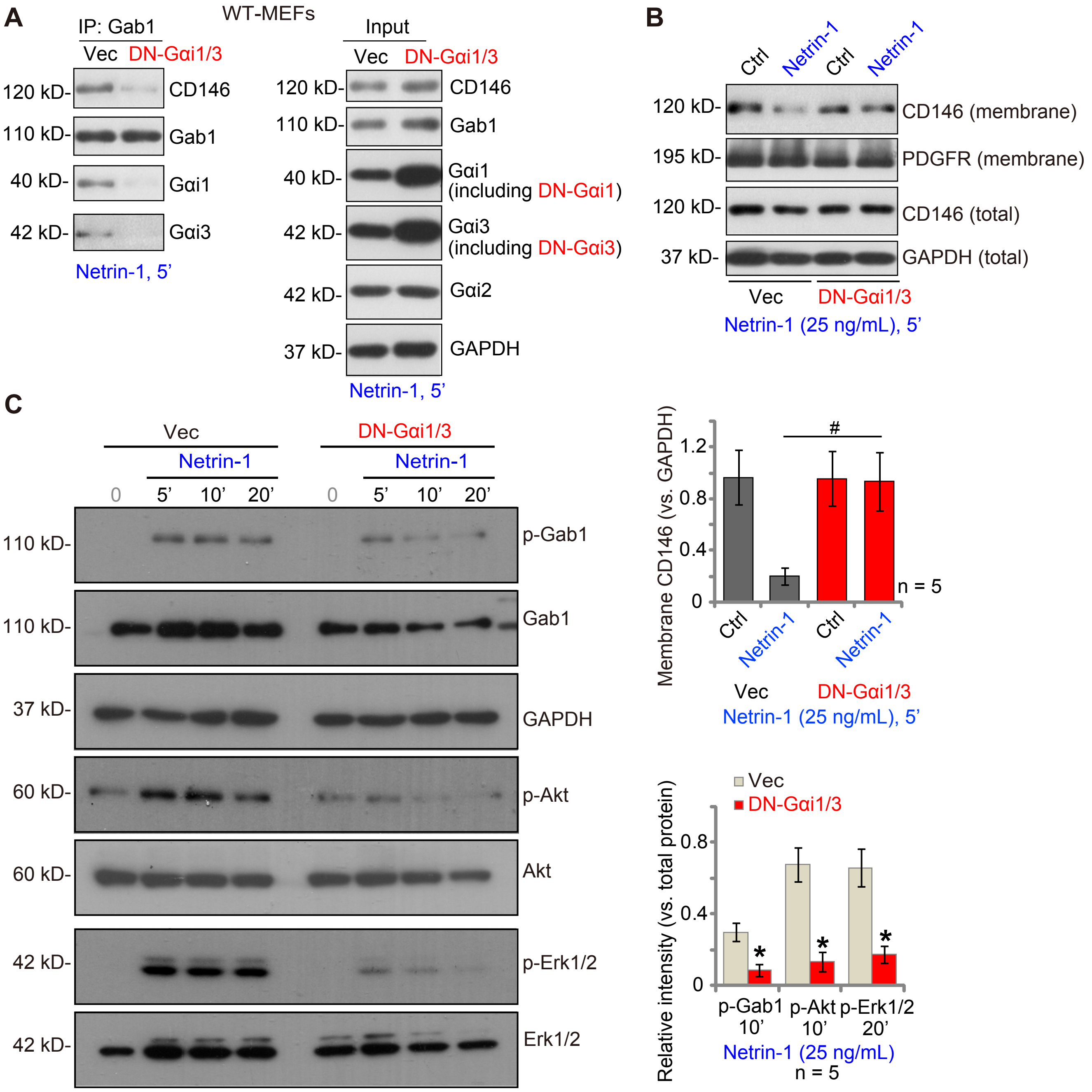

A
WT-MEFs
IP: Gab1
Vec DN-Gαi1/3
120 kD- CD146
110 kD- Gab1
40 kD- Gαi1
42 kD- Gαi3
Netrin-1, 5'
Input
Vec DN-Gαi1/3
120 kD- CD146
110 kD- Gab1
40 kD- Gαi1 (including DN-Gαi1)
42 kD- Gαi3 (including DN-Gαi3)
42 kD- Gαi2
37 kD- GAPDH
Netrin-1, 5'
B
Ctrl Netrin-1 Ctrl Netrin-1
120 kD- CD146 (membrane)
195 kD- PDGFR (membrane)
120 kD- CD146 (total)
37 kD- GAPDH (total)
Vec DN-Gαi1/3
Netrin-1 (25 ng/mL), 5'
Membrane CD146 (vs. GAPDH)
1.2
0.8
0.4
0
#
n = 5
Ctrl Netrin-1 Ctrl Netrin-1
Vec DN-Gαi1/3
Netrin-1 (25 ng/mL), 5'
C
Vec
DN-Gαi1/3
Netrin-1
Netrin-1
0 5' 10' 20' 0 5' 10' 20'
110 kD- p-Gab1
110 kD- Gab1
37 kD- GAPDH
60 kD- p-Akt
60 kD- Akt
42 kD- p-Erk1/2
42 kD- Erk1/2
Vec
DN-Gαi1/3
Relative intensity (vs. total protein)
0.8
0.4
0
*
p-Gab1 10'
p-Akt 10'
p-Erk1/2 20'
Netrin-1 (25 ng/mL)
n = 5

Figure S3. Gαi1/3 dominant negative mutants disrupt Netrin-1-induced CD146-Gαi1/3-Gab1 association, CD146 internalization and signaling activation. Puromycin-selected WT MEFs, with the dominant negative mutant-Gαi1 plus the dominant negative mutant-Gαi3 ("DN-Gαi1/3") or the empty vector ("Vec"), were treated with Netrin-1 (25 ng/mL) for 5 min, CD146-Gαi1/3-Gab1 association ("IP") and expression ("Input") was shown (**A**); Expression of the listed proteins in membrane fraction lysates and total cell lysates was also shown (**B**). Netrin-1 (25 ng/mL)-activated signalings were tested as well (**C**), and protein phosphorylation was quantified (**C**). Quantifications were from five biological replicates (n = 5). $^{*}P < 0.05$ versus "Vec" cells. $^{\#}P < 0.05$.

Figure S4

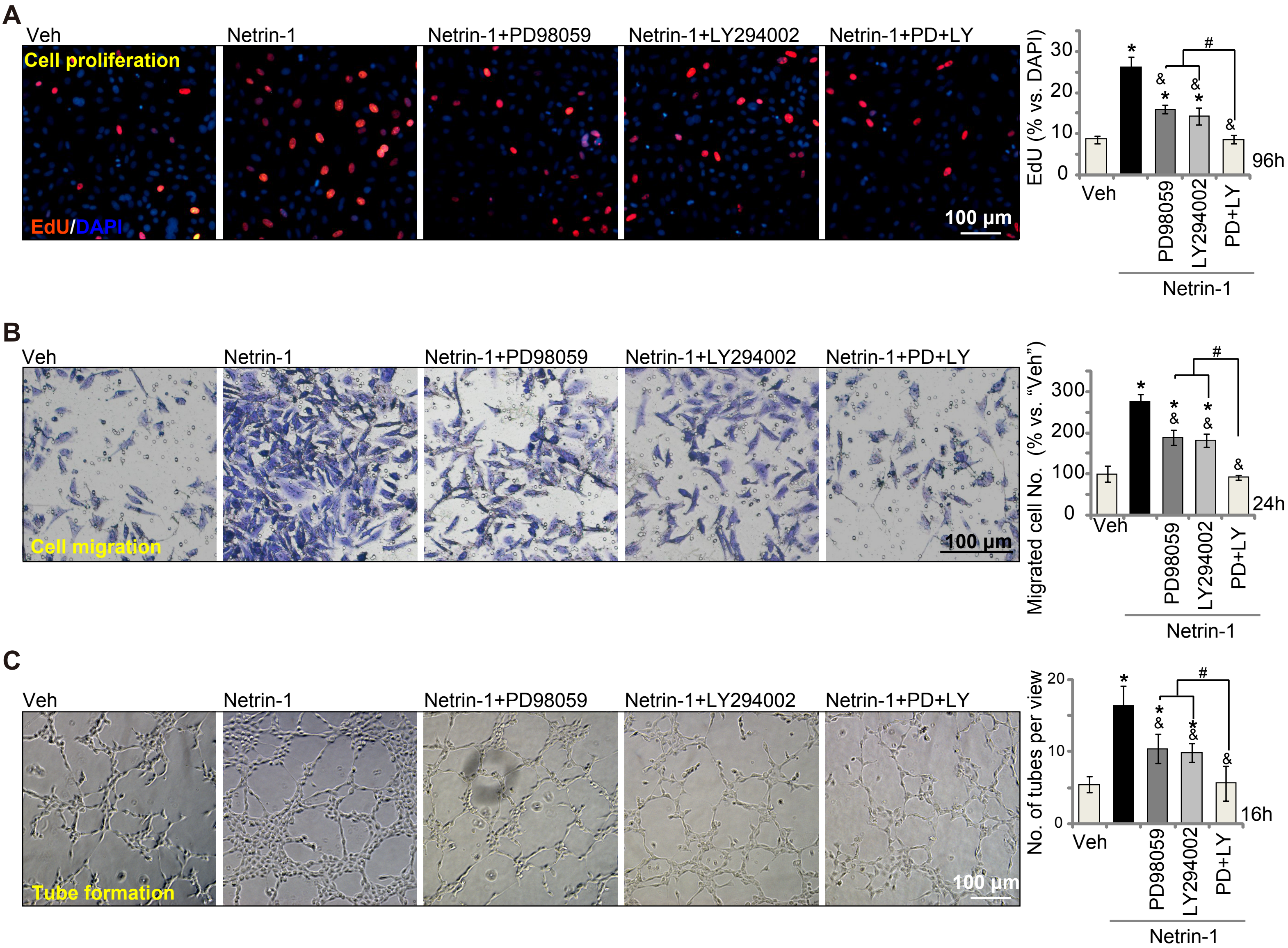

A
Veh
Netrin-1
Netrin-1+PD98059
Netrin-1+LY294002
Netrin-1+PD+LY
Cell proliferation
EdU/DAPI
100 μm
EdU (% vs. DAPI)
30
20
10
0
96h
Veh
PD98059
LY294002
PD+LY
Netrin-1
B
Veh
Netrin-1
Netrin-1+PD98059
Netrin-1+LY294002
Netrin-1+PD+LY
Cell migration
100 μm
Migrated cell No. (% vs. "Veh")
300
200
100
0
24h
Veh
PD98059
LY294002
PD+LY
Netrin-1
C
Veh
Netrin-1
Netrin-1+PD98059
Netrin-1+LY294002
Netrin-1+PD+LY
Tube formation
100 μm
No. of tubes per view
20
10
0
16h
Veh
PD98059
LY294002
PD+LY
Netrin-1

Figure S4. Erk and PI3K-Akt-mTOR inhibition suppresses Netrin-1-induced pro-angiogenic activity in HUVECs. HUVECs were pretreated with the Erk inhibitor PD98059 (5 μ M), the PI3K-Akt-mTOR inhibitor LY294002 (5 μ M) or PD98059 plus LY294002 ("PD+LY") for 1h, followed by Netrin-1 (25 ng/mL) treatment, HUVECs were cultivated for applied time periods, cell proliferation (by measuring EdU incorporation, **A**), migration (**B**), and tube formation (**C**) were tested. The data were presented as mean ± standard deviation (SD, n = 5, five biological replicates). "Veh" stands for vehicle control group. * $P < 0.05$ versus "Veh". $^{\&}$ $P < 0.05$ versus Netrin-1 only treatment. $^{\#}P < 0.05$. Scale bar = 100 μm.
